# Design of Epitope-Focused Vaccines via Epitope Cleavage

**DOI:** 10.1021/acscentsci.6c00476

**Published:** 2026-07-17

**Authors:** Dominic M. Pham, Rebekah M. Costello, Theodora U. J. Bruun, Peter S. Kim

**Affiliations:** † Stanford Biophysics Program, 10624 Stanford University School of Medicine, Stanford, California 94305, United States; ‡ Sarafan ChEM-H, 6429 Stanford University, Stanford, California 94305, United States; § Program in Immunology, Stanford University School of Medicine, Stanford, California 94305, United States; ∥ Department of Biochemistry, Stanford University School of Medicine, Stanford, California 94305, United States

## Abstract

The design of epitope-focused immunogens that steer serum antibody responses toward epitopes targeted by broadly neutralizing antibodies (bnAbs) is a promising approach to develop broad-spectrum vaccines against pathogens with high genetic variability. Here, we introduce a strategy called epitope cleavage, which involves “cleaving” an epitope to steer antibodies away from it onto other regions of an antigen. We demonstrated the utility of epitope cleavage using two independent approaches: circular permutation and proteolytic cleavage. We first designed a sarbecovirus vaccine candidate by circularly permuting the receptor binding domain (RBD) of the severe acute respiratory syndrome coronavirus 2 (SARS-CoV-2) spike protein; we next designed broad Ebolavirus immunogens by inserting the TEV protease cleavage site into the glycan cap of Ebola glycoprotein (GP) for site-specific epitope cleavage. By cleaving variable regions on each antigen, both the circular permuted RBD and cleaved GPs elicited antibodies with enhanced cross-reactivity to related viruses compared to unmodified antigen and exhibited a reduction in antibody responses toward the “cleaved” epitope. Our results present epitope cleavage as a vaccine design strategy that redirects antibody responses without compromising immunogenicity.

## Graphical abstract

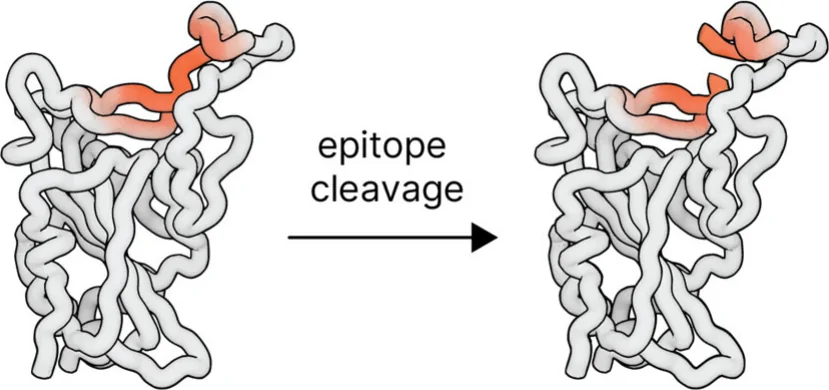

## Introduction

A major hurdle in vaccine development is that responses to vaccines against pathogens with high sequence variability are often strain-specific.^1–3^ Immunofocusing strategies aim to provide broad protection against a group of related pathogens by directing the humoral immune response toward conserved regions of a virus.^4,5^ Existing methods for creating such epitope-focused vaccines vary in how precisely one can control the antibody response against a given antigen. Since bnAbs and other undesirable antibodies can share overlapping epitopes on an antigen,^5–9^ the design of broad-spectrum vaccines may ultimately benefit from immunofocusing methods that are site-specific, allowing for precise tuning of an epitope’s immunogenicity.

Many established site-specific immunofocusing techniques are based on the concept of epitope masking, where modifications are added onto immunogens in a site-directed manner to sterically block select epitopes. Notable examples include hyperglycosylation—which uses endogenous mammalian glycosylation machinery to create a glycan shield around undesirable epitopes and has resulted in broad HIV, SARS-CoV-2, influenza, Zika virus and Ebolavirus vaccine candidates^10–15^ —and chemical modification with bulky polyethylene glycol (PEG) moieties—which has been used to engineer influenza and SARS-CoV-2 immunogens.^16,17^ However, one drawback of these epitope masking approaches is that they can decrease the overall immunogenicity of the immunogens, resulting in lower overall antibody titers and requiring multiple boosts to rescue immunogenicity.^12,14,17–20^ Thus, there is a need to develop new immunofocusing techniques that (1) perturb antigenicity and immunogenicity in an epitope-specific manner and (2) do not dampen the overall immunogenicity of engineered immunogens.

Here, we introduce a new concept in immunofocusing called *epitope cleavage*. This approach draws inspiration from the viral immune-evasion strategy of entropic masking, where highly flexible regions of an antigen pose an entropic penalty on antibodies that bring them into order upon binding.^21,22^ Given that rigidification or stabilization of epitopes can improve their antigenicity and/or immunogenicity,^23–25^ we surmised that selective modulation of a given epitope’s flexibility may alter its immunogenicity. By extension, we hypothesize that breaking or cleaving an off-target epitope can reduce its immunogenicity by disrupting its structural integrity to create a hyperflexible region, making it more difficult for B-cell receptors to recognize the cleaved epitope within the immunogen. We thus set out to design immunofocusing methods that cleave epitopes in a site-specific manner and do not perturb the antigenicity of off-target epitopes.

We present two approaches for epitope cleavage: circular permutation and proteolytic cleavage. Circular permutation is a rearrangement of the primary sequence of a protein to introduce new internal termini by cleaving an existing bond and fusing the native termini of a protein together.^26^ Although circular permutation has been used in vaccine design previously to modulate the multimeric state of an antigen^27,28^ and for multimeric display of antigens,^29^ here we harness circular permutation to demonstrate the potential of epitope cleavage. By engineering new termini in the middle of a variable epitope (Figure 1a), we hypothesize that this effectively “cleaves” the epitope and redirects antibody responses away from it. We use circular permutation to design a vaccine candidate based on the SARS-CoV-2 receptor binding domain (RBD) to serve as a sarbecovirus vaccine candidate. Our results demonstrate that the circularly permuted RBD (RBDcp) elicited antibodies with enhanced cross-reactivity to sarbecoviruses from all three clades and focused the immune response onto conserved epitopes compared to wildtype RBD.

**Figure 1:**
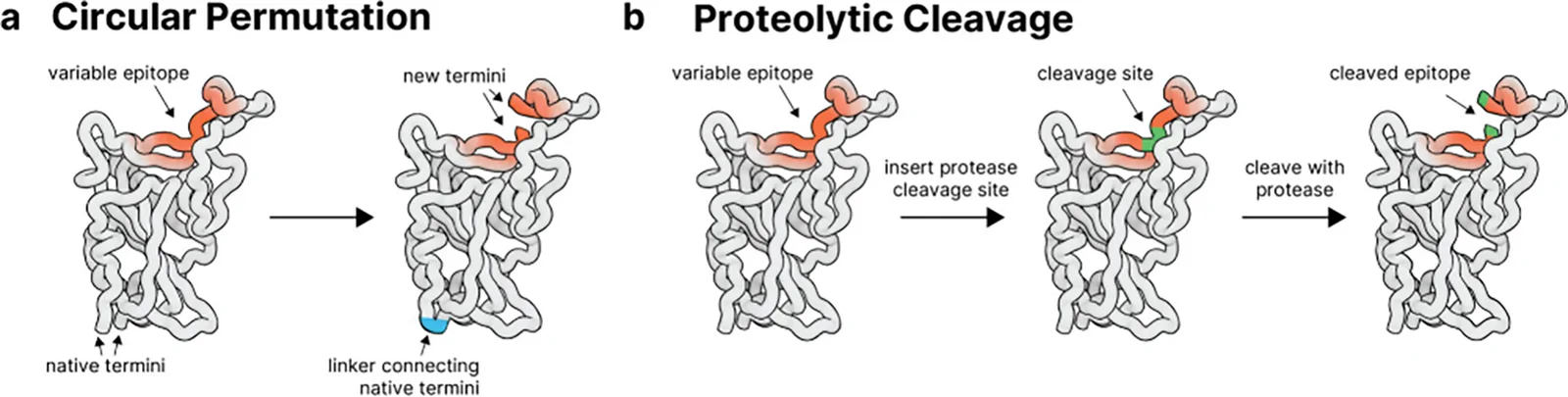
Two protein engineering techniques for epitope cleavage. (a) Circular permutation consists of genetically fusing the native termini of a protein with a linker (blue) and then generating new termini at another site in the protein. (b) Proteolytic cleavage involves genetically inserting a protease recognition site (green) into a variable epitope and then cleaving with the cognate protease.

Next, we employed proteolytic cleavage as a technique for epitope cleavage by inserting a protease cleavage site within an epitope (Figure 1b). We cleaved sites of the variable glycan cap region of Ebola glycoprotein (GP) with TEV protease to design two universal Ebolavirus vaccine candidates, showing that site-specific proteolytic cleavage selectively disrupted antibody binding to cleaved immunogens. Compared to unmodified GP, mice immunized with cleaved GPs show greater cross-reactivity to all six known Ebolavirus species and robust cross-neutralization of all three Ebolavirus species that have caused outbreaks in humans: EBOV, SUDV, and BDBV.^30^ Our results present epitope cleavage as a promising site-directed approach to epitope-focused vaccine design.

## Results

### Epitope Cleavage via Circular Permutation of the SARS-CoV-2 Receptor Binding Domain

In order to design a SARS-CoV-2 RBD-based immunogen that could elicit broad humoral immune responses to related sarbecoviruses, we aimed to “cleave” the hypervariable receptor binding motif (RBM), a region of the RBD responsible for binding to its receptor ACE2.^31^ We circularly permuted the RBD sequence by genetically fusing the native N- and C-termini of the RBD (residues 319 and 533) with a (GS)_4_ linker and scanned through residues 475–503 to find places to install new termini and “cleave” this region. After expressing each circular permutant in mammalian cells and probing the supernatant for expression via dot blot, we identified a circular permutant with new termini at residues 487 and 488, hereon referred to as RBDcp (Figure 2a and Figure S1a). This variant showed the highest level of expression in mammalian cells out of all constructs tested and yielded a similar SEC elution profile as wild-type RBD (RBDwt) after Ni-NTA affinity purification with a C-terminal 6xHis tag on both RBDwt and RBDcp (Figure 2b,c). Thermal melt measurements indicated that RBDcp has a similar thermostability to RBDwt with a melting temperature around 53 °C (Figure 2d). Since RBDcp has its termini in an immunodominant, variable epitope to which strain-specific class 1 and 2 antibodies bind,^32,33^ we hypothesized that this construct may be a suitable immunogen to serve as a broad sarbecovirus vaccine candidate by immunofocusing the antibody response away from the RBM and onto more conserved epitopes like the cryptic face.^34^ We then probed the antigenicity of RBDcp using a panel of RBD-targeted antibodies from five distinct classes: class 1 (CB6), class 2 (C002), class 3 (SA58), class 4 (S2X259), and class 1/4 (SA55). While RBDcp showed a similar EC_50_ to SA58, S2X259, and SA55 via ELISA, binding to CB6 was over 1000-fold worse and binding to C002 was completely ablated (Figure 2e,f). Similarly, we observed loss of binding to RBM-directed binders in BLI experiments, where RBDcp showed minimal binding to CB6, C002, and ACE2-Fc while maintaining binding to SA58, S2X259, and SA55 (Figure S1b). These data indicate that RBDcp introduces a local structural perturbation at the new termini while maintaining the thermostability of the protein and antigenicity of off-target epitopes.

**Figure 2:**
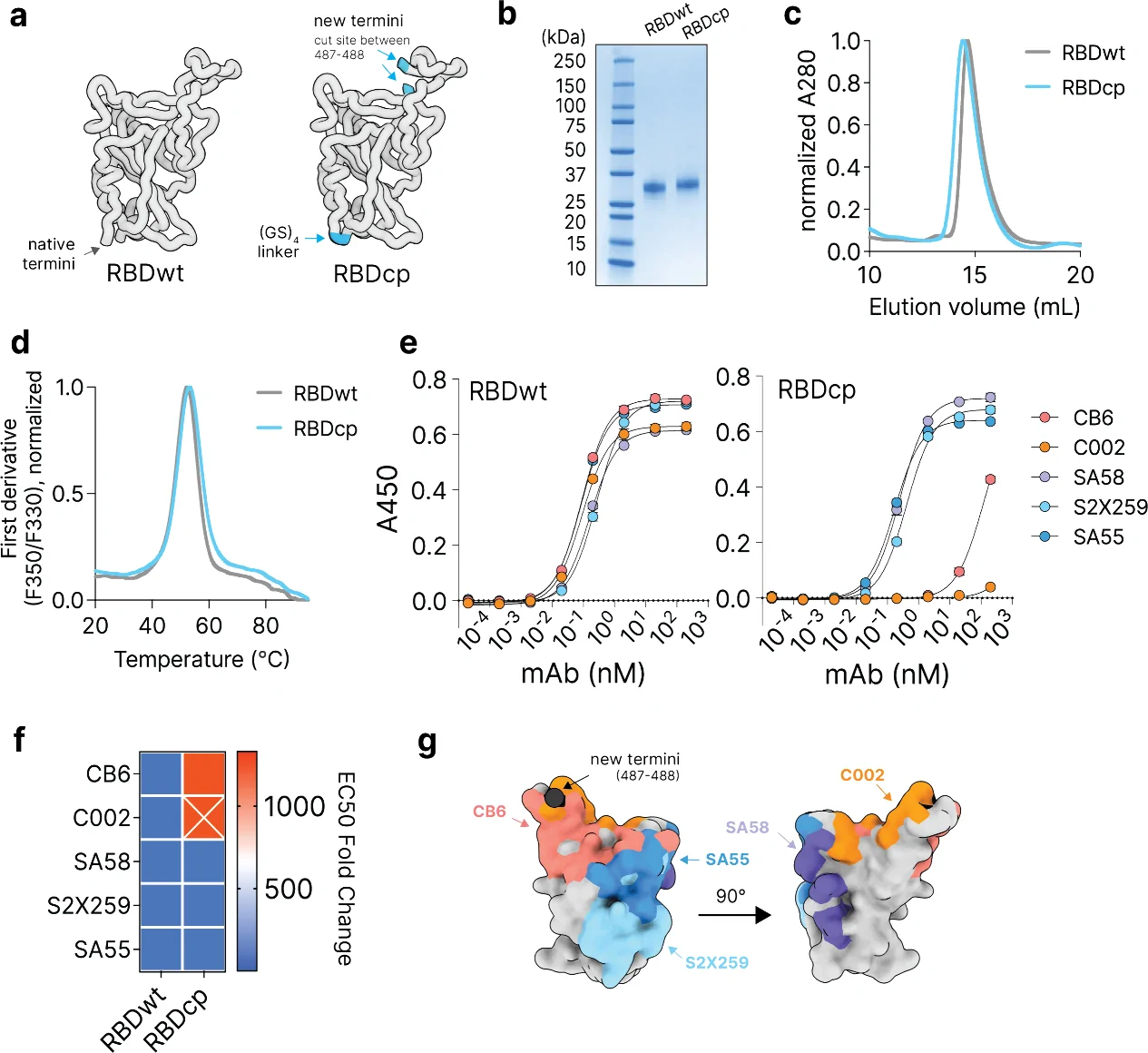
Circular permutation of SARS-CoV-2 RBD. (a) Structural representation of SARS-CoV-2 RBD (PDB ID: 6M0J, gray) and a circularly permuted variant between residues 487–488. (b) Gel electrophoresis of wild-type and circularly permuted RBD. Gel was stained with Coomassie brilliant blue. (c) Size-exclusion purification of RBDwt and RBDcp. (d) Representative thermal melting profiles of RBDwt and RBDcp measured by differential scanning fluorimetry (*n* = 3 technical replicates). (e) ELISA for epitope analysis of RBDwt and RBDcp with five RBD-specific mAbs. Binding data are presented as mean ± SD (*n* = 3 technical replicates). (f) Fold-change in binding (EC_50_) of RBD-specific mAbs (columns) to RBDwt and RBDcp (rows). EC_50_ (antibody concentration with half-maximal binding) was calculated from (e) and normalized to values obtained from the RBDwt group. A crossed-out box in red indicates that an EC_50_ could not be calculated for the corresponding curve. (g) Structure of RBD (PDB ID: 6M0J, gray) with five epitopes of five monoclonal antibodies used for ELISA binding experiments in different colors and the site for circular permutation highlighted (dark gray).

### RBDcp Elicits a Broad, Pan-Sarbecovirus Antibody Response Targeting Conserved Epitopes

To test whether RBDcp could serve as a broad sarbecovirus vaccine candidate, we intramuscularly immunized mice with either RBDwt or RBDcp adjuvanted with MPLA/QuilA (Figure 3a). We found that throughout the course of the experiment, RBDcp induced similar serum IgG titers against SARS-CoV-2 RBD and full-length spike when compared to RBDwt, demonstrating that circular permutation did not dampen the immunogenicity of RBD (Figure 3b and Figure S2a).

**Figure 3:**
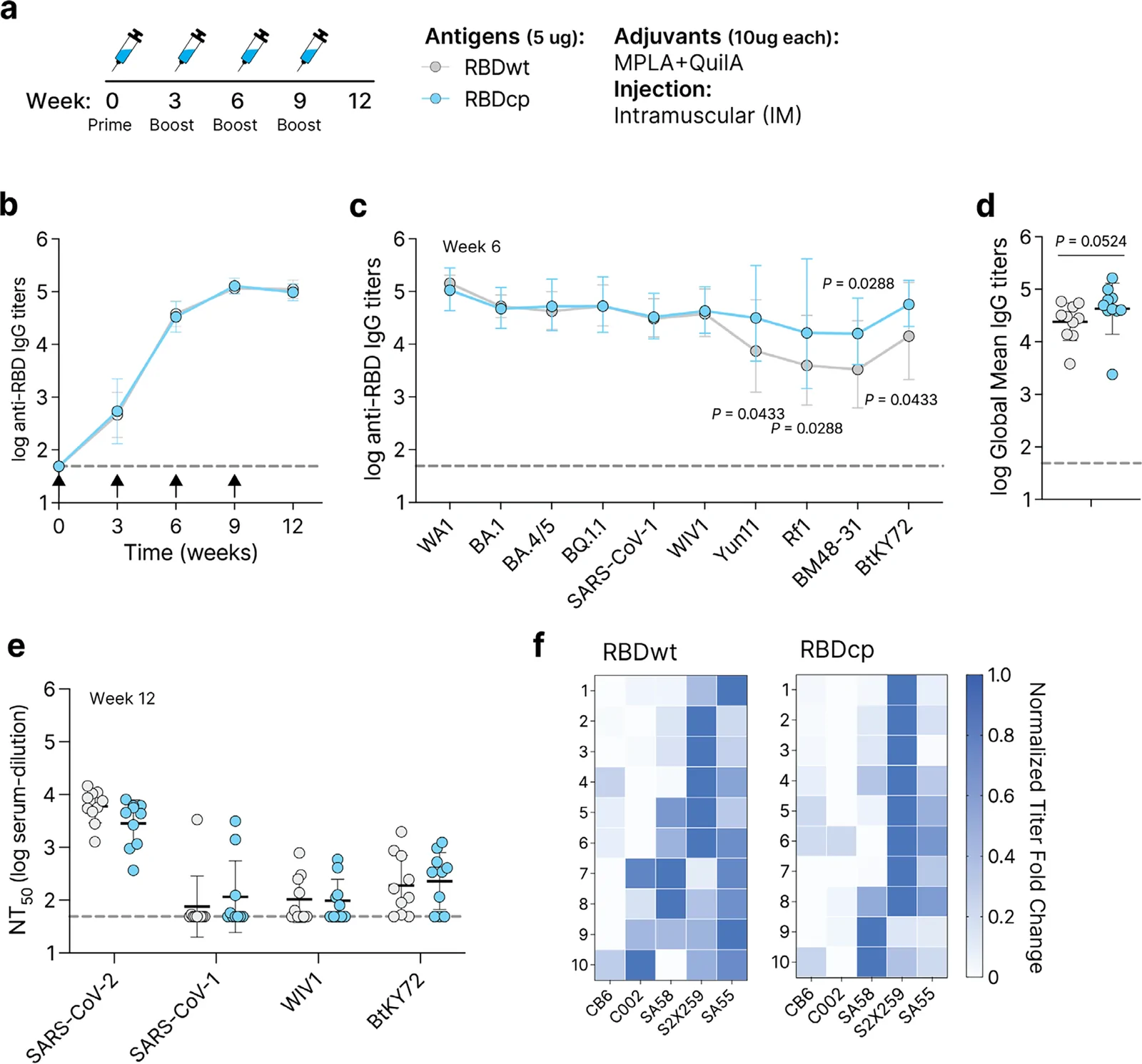
RBDcp induces a cross-reactive antibody response targeting conserved epitopes on the RBD. (a) A four-dose immunization study with RBDwt or RBDcp adjuvanted with MPLA and QuilA via intramuscular injection in mice. (b) Serum IgG titers against SARS-CoV-2 RBD over time. Arrows indicate prime immunization followed by three boosts. Each circle represents the geometric mean IgG titer per group. (c) IgG titers against SARS-CoV-2 variants (WA1, BA.1, BA.4/5, BQ.1.1), clade 1a (SARS-CoV-1 and WIV1), clade 2 (Yun11 and Rf1), and clade 3 (BM48-31 and BtKY72) sarbecovirus RBDs by week 6 antisera. Each circle represents the geometric mean IgG titer per group. (d) Global geometric mean titers against RBDs tested at week 6. Each circle represents a single mouse (*n* = 10 mice per group). (e) Neutralization titers against SARS-CoV-2 WA1, SARS-Cov-1, WIV1, and BtKY72 (K493Y/T498W) pseudotyped viruses by week 12 antisera. (f) Immunodominance hierarchies^35^ of each individual mouse from competition ELISA with week 6 serum. Each titer fold-change from blocking with RBD-specific mAbs (columns) was normalized from zero to one for each mouse (rows) immunized with RBDwt (left) and RBDcp (right).

We next tested the cross-reactivity of the antisera from each group to a panel of RBDs from diverse sarbecorviruses from all three major clades. Antibody titers from mice immunized twice with RBDcp were significantly higher against RBDs from sarbecoviruses from clades 2 and 3 (Yun11, Rf1, BM48-31, and BtKY72) compared to RBDwt and similar against clade 1a sarbecovirus RBDs (SARS-CoV-1 and WIV1) and SARS-CoV-2 variants (BA.1, BA.4/5, and BQ.1.1) (Figure 3c and Figure S2b). Furthermore, we observed this enhancement in cross-reactivity toward clade 2 and/or clade 3 sarbecoviruses throughout the course of the mouse experiment (Figure S2f–h).

When testing the neutralization activity of the antisera, we found that the mice immunized with RBDcp had slightly lower neutralization titers against SARS-CoV-2 WA.1 compared to RBDwt after a prime and three boosts (Figure 3e and Figure S3a), although this difference was not statistically significant. This difference in neutralization activity may reflect how some of the most potently neutralizing antibodies against SARS-CoV-2 are strain-specific and target class 1 and 2 epitopes, while antibodies with more breadth targeting class 3 and 4 epitopes are less potently neutralizing.^34^ However, when testing the cross-neutralization of the serum against clade 1a (SARS-CoV-1 and WIV1) and clade 3 sarbecoviruses (BtKY72), the RBDcp group showed comparable cross-neutralization to the RBDwt group (Figure 3e and Figure S3b–d).

To further evaluate the basis of this cross-reactivity and to determine the immunodominance hierarchy^35^ of the antisera, we then performed competition ELISA with a representative antibody from classes 1, 2, 3, 4 and 1/4. Since we tested competition by blocking with antibodies from five major classes of RBD-targeting antibodies covering a major portion of the antigenic landscape of the RBD (Figure 2g),^33,34^ we constructed immunodominance hierarchies from each mouse from the competition ELISA data.^35^ We calculated titer fold changes for each mouse in response to blocking with CB6, C002, SA58, S2X259, and SA55 (Figure S2c–e) and normalized the log-transformed titer fold changes for each mouse. Mice immunized with RBDwt showed an inconsistent mixture of immunodominance hierarchies, which may reflect how all epitopes are equally exposed when the RBD is administered as a recombinant protein (Figure 3f). On the contrary, mice immunized with RBDcp showed a more consistent pattern of immunodominance. Eight mice immunized with RBDcp exhibited an immunodominance hierarchy focused on S2X259, a class 4 antibody that targets a conserved epitope among sarbecoviruses but shows limited breadth against BQ.1.1 and XBB variants of SARS-CoV-2;^36,37^ the other two mice showed an immunodominant response to the class 3 antibody SA58 (Figure 3f and Figure S2c–e). These results indicate that RBDcp shifted the antibody response away from the class 1 and 2 epitopes on the RBM toward more conserved epitopes. Taken together, these data suggest that RBDcp successfully elicited an enhanced cross-reactive antibody response toward diverse sarbecoviruses without dampening the overall immunogenicity of the antigen. Although antibodies induced by RBDcp are less potent neutralizers of SARS-CoV-2, they neutralize sarbecoviruses from other clades with similar or enhanced activity compared to that of RBDwt. Furthermore, the enhanced cross-reactive binding titers in mice immunized with RBDcp could play an important role in protection against pandemic potential sarbecoviruses, as non-neutralizing antibodies against SARS-CoV-2 are thought to confer protection via Fc effector functions.^38^

### Proteolytic Cleavage Achieves Precise Epitope Knockout on Ebola Glycoprotein

Inspired by the findings with RBDcp, we next sought to create an orthogonal technique to circular permutation for epitope cleavage, using EBOV GP and TEV protease as a model to directly cleave epitopes with proteases. Since we wanted to cleave EBOV GP at non-native sites and to ensure that TEV protease would cleave the constructs into two fragments, we first mutated the native furin cleavage site from RRTRR to AGTAA to abolish furin cleavage^39,40^ (Δfurin). Using a design that included GP with its mucin-like domain removed (ΔMLD) as the background, we confirmed that mutating the furin cleavage site (GPΔMLDΔfurin) did not affect the size, thermostability, and antigenicity of GP (Figure S4b–e). Furthermore, we found that under reducing conditions, purified GPΔMLD was cleaved into two fragments (GP1 and GP2) while GPΔMLDΔfurin was not cleaved (Figure S4a). Hereafter, we refer to GPΔMLDΔfurin as GPwt.

We then aimed to use TEV protease to cleave the variable glycan cap of EBOV GP, following a similar approach to a study that used hyperglycosylation to mask the glycan cap.^12^ We first investigated whether GP could tolerate insertion of the TEV protease cleavage site (TEV_CS_), a seven amino acid sequence (ENLYFQS), into glycan cap loops. We screened 11 insertion sites in multiple glycan cap loops, and we determined via dot bot that inserting the TEV_CS_ in each site yielded protein that expressed in mammalian cells (Figure S5a), demonstrating that EBOV GP was readily able to accommodate insertion of the TEV_CS_ into different loops on the glycan cap. We then selected two insertion sites in distinct regions of the variable glycan cap to perform further analysis on R266 at the top and F290 on the side of the glycan cap (Figure 4a); these sites were chosen based on their expression levels (Figure S5a) and ability of TEV protease to cleave at these sites. For the insertion site at R266, we noticed that simple insertion of the TEV_CS_ only allowed the TEV protease to partially cleave the GP. Therefore, we flanked the TEV_CS_ insertion on both sides with (GS)_2_ to improve accessibility of the site and cleavage efficiency (Figure S5b) and used this construct in subsequent experiments.

**Figure 4:**
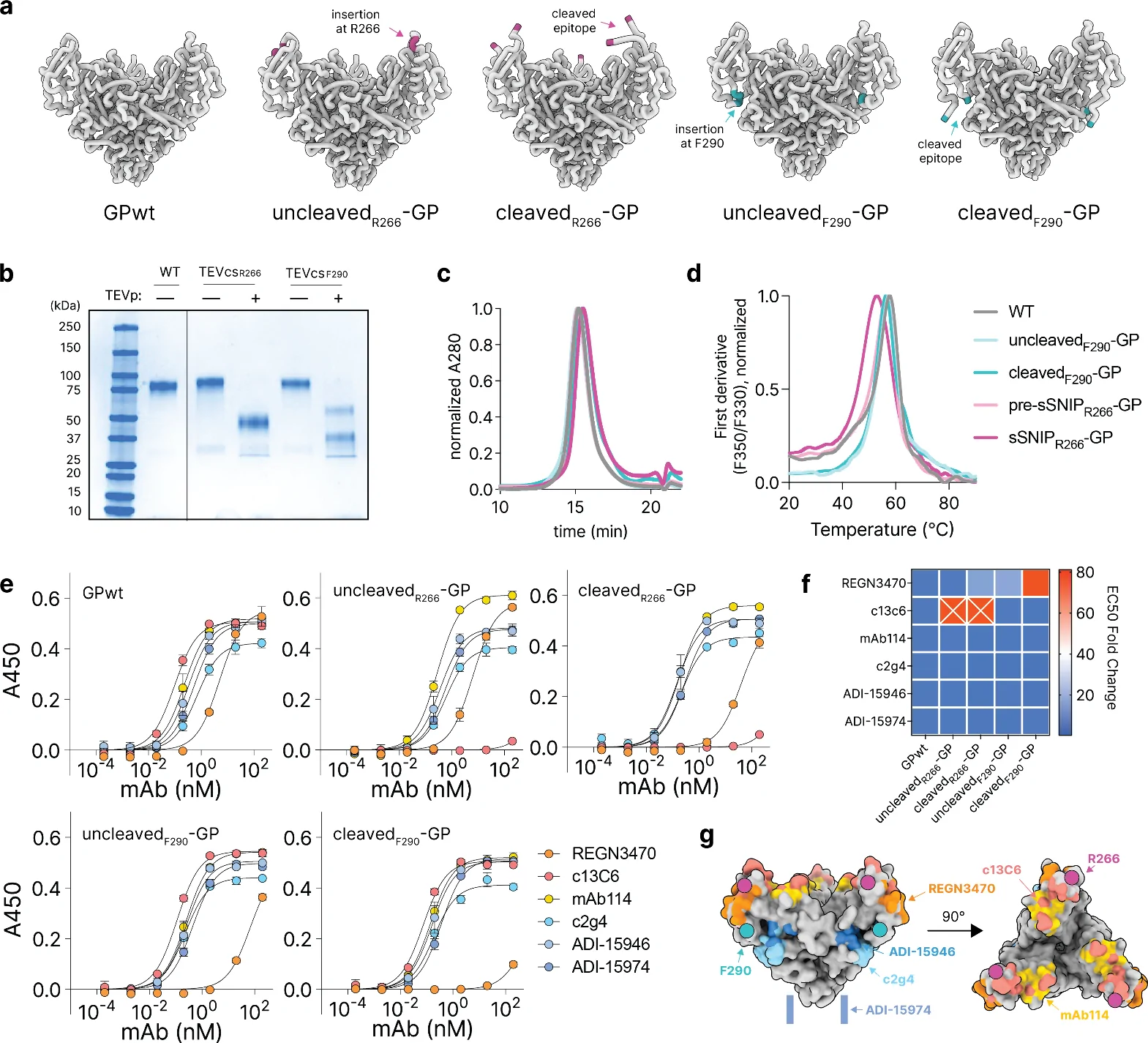
Site-specific proteolytic cleavage for precise epitope knockout on Ebola glycoprotein. (a) Structural representation of EBOV GP (PDB ID: 7TN9, gray) with two TEV_CS_ insertion sites on the glycan cap. Uncleaved GPs include the TEV_CS_ insertion but were not treated with TEV protease, while cleaved GPs have been cleaved by TEV protease. (b) Gel electrophoresis of GP constructs. Gel was run under reducing conditions and stained with Coomassie brilliant blue. (c) SEC traces of GP constructs. (d) Representative melting curves of GP constructs measured by differential scanning fluorimetry (*n* = 3 technical replicates). (e) ELISA for epitope analysis of GP constructs with six GP-specific mAbs. Binding data are presented as mean ± SD (*n* = 3 technical replicates). (f) Fold-change in binding (EC_50_) of GP-specific mAbs (columns) to GP variants (rows). EC_50_ (antibody concentration with half-maximal binding) was calculated from (e) and normalized to values obtained from the GPwt group. A crossed-out box indicates that an EC_50_ could not be constructed for the corresponding curve. (g) Structure of EBOV GP (PDB ID: 7TN9, gray) with six epitopes of six monoclonal antibodies used for ELISA binding experiments in different colors and two sites for cleavage highlighted (magenta and teal).

We confirmed cleavage of the GPs through a reducing gel on constructs before (uncleaved) and after (cleaved) TEV protease digestion (Figure 4b). TEV protease split cleaved _R266_-GP into two fragments with similar masses, indicated by the formation of a lower band on the gel, while cleaved_F290_-GP was cleaved with two fragments around 37 kDa and above 50 kDa (Figure 4b). We then performed size exclusion chromatography coupled with multiangle light scattering (SEC-MALS) to determine if cleavage affected the ability of GP to form intact trimers. The SEC traces indicated that cleavage did not perturb the multimeric state of GP since all constructs eluted around similar times (Figure 4c), and all constructs had a mass around 240 kDa, indicating that all proteins primarily formed trimers (Figure S6b,c). We then measured the thermostability of each immunogen, finding that while cleaved _R266_-GP had a slightly lower Tm than the other constructs by 4 °C, all proteins were stable at physiological temperature (37 °C) (Figure 4d and Figure S6a). These data demonstrate that we were able to efficiently cleave EBOV GP with TEV protease at two independent sites on the glycan cap without significantly disturbing the multimeric state or thermostability compared to GPwt.

To examine how efficiently proteolytic cleavage perturbed epitopes on GP, we tested binding to a panel of six GP-targeting mAbs that recognize diverse epitopes spanning the glycan cap/head domain (REGN3470, c13C6, and mAb114), the base (ADI-15946 and c2g4), and the stalk (ADI-15974) of GP. Uncleaved_F290_-GP showed a decrease in binding to REGN3470 compared to GPwt, but cleaved_F290_-GP ablated binding even further (Figure 4e,f). TEV protease cleavage at F290 results in an 80-fold difference in EC_50_ compared to GPwt, demonstrating that cleavage of this loop allosterically disrupts binding of this glycan-cap-targeted antibody. Similarly, since the loop containing residue R266 is directly adjacent to the REGN3470 epitope,^41^ cleaved_R266_-GP exhibited a decrease in binding to REGN3470 compared to uncleaved_R266_-GP and GPwt. In addition, we found that insertion of the TEV_CS_ after R266 (uncleaved_R266_-GP) itself was sufficient to knockout binding of the glycan cap binder c13C6, and cleaved_R266_-GP retained this loss of binding (Figure 4e,f). Furthermore, epitope cleavage did not perturb off-target epitopes as binding of mAb114, c2g4, ADI-15946, and ADI-15794 were retained. These data suggest that proteolytic cleavage selectively ablated binding of antibodies directed at cleaved epitopes.

### Cleaved GPs Elicit Enhanced Cross-Reactivity against Diverse Ebolavirus Species

To compare the immunogenicity of cleaved GPs to unmodified GP (GPwt), we intramuscularly administered mice in five groups with GPwt, uncleaved_R266_-GP, cleaved_R266_-GP, uncleaved_F290_-GP, or cleaved_F290_-GP adjuvanted with AddaS03 (Figure 5a and Figure S7a). We found that proteolytic cleavage maintains the immunogenicity of GP, as there were equivalent antibody titers against EBOV GP between both groups throughout the experiment (Figure 5b). When profiling cross-reactive binding to other Ebolavirus species, cleaved_F290_-GP induced significantly higher antibody titers against all five other Ebolavirus GPs compared to GPwt, and mice immunized with cleaved_R266_-GP induced significantly higher titers against all but BOMV GP (Figure 5c), although some of these increases are modest. Mice immunized with both cleaved GPs exhibited higher levels of cross-reactivity compared to their uncleaved counterparts, while the uncleaved GPs induced similar antibody titers to GPwt in many cases (Figure S7b,c). The global mean antibody titer across all Ebolavirus species was significantly higher in mice immunized with both cleaved GPs compared to GPwt and their uncleaved counterparts (Figure 5d and Figure S7f). These data suggest that simple insertion of TEV_CS_ does not substantially alter the immunogenicity of GP and that TEV protease cleavage was necessary to observe enhanced cross-reactivity.

**Figure 5:**
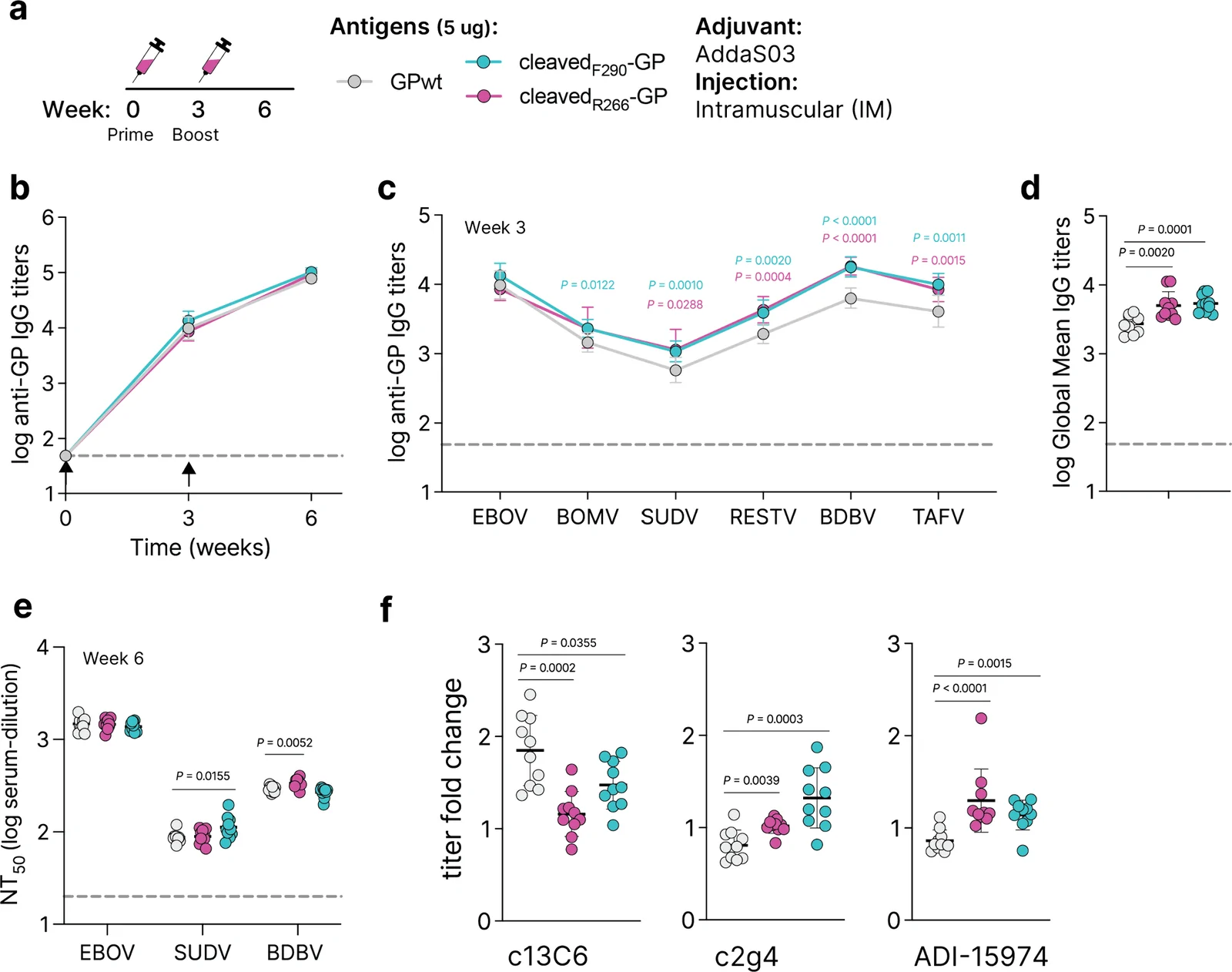
Cleaved GPs induce a more cross-reactive antibody response to GPs from all six Ebolavirus species. (a) A two-dose immunization study with GPwt, cleaved_R266_-GP, and cleaved_F290_-GP adjuvanted with AddaS03 via intramuscular injection in mice. (b) Serum IgG titers against GPwt over time. Arrows indicate prime immunization followed by a boost. Each circle represents the geometric mean IgG titer per group. (c) IgG binding titers against GPs from all six Ebolavirus species (EBOV, BOMV, SUDV, RESTV, BDBV and TAFV) by week 3 antisera. Each circle represents the geometric mean IgG titer per group. (d) Global geometric mean titers against all 6 GPs tested at week 3. Each circle represents a single mouse (*n* = 10 mice per group). (e) Neutralization titers against EBOV, SUDV and BDBV pseudotyped viruses by week 6 antisera. (f) Titer fold-changes from competition ELISA with week 6 serum in the presence of competing mAbs (c13C6, c2g4, and ADI-15974). Each circle represents the titer fold-change from a single mouse (*n* = 10 mice per group).

We next investigated the neutralization activity of elicited antibodies against all three species of Ebolavirus which have caused multiple outbreaks in humans: EBOV, SUDV, and BDBV.^30^ All groups showed similar pseudovirus neutralization titers against EBOV, further demonstrating that proteolytic cleavage did not alter the immunogenicity of GP (Figure 5e and Figure S8a,b). Furthermore, cleaved_F290_-GP and cleaved_R266_-GP induced statistically higher neutralization titers against SUDV and BDBV respectively compared to GPwt, although these differences are subtle (Figure 5e and Figure S8). We directly show that epitope cleavage—and not simple insertion of the TEV_CS_—may slightly alter the profile of the antisera, as uncleaved GPs induced similar neutralization titers to GPwt (Figure S8a).

In order to explore the basis of this enhanced cross-reactivity and cross-neutralization and to evaluate the extent of immunofocusing, we performed competition ELISA with mAbs targeting the glycan cap, base, and stalk epitopes of GP. Antibodies induced by cleaved GPs showed less competition with the glycan cap-targeted antibody c13C6 and more competition with base (c2g4) and stalk (ADI-15974) antibodies compared to GPwt, as indicated by shifts in the titer fold change after blocking with these antibodies (Figure 5f and Figure S7d). When testing the competition profile of antibodies elicited by uncleaved GPs, these groups showed a reduced effect compared to antibodies from cleaved GPs. Both cleaved GPs elicited less glycan-cap-competing antibodies (although not significant) and significantly higher amounts of stalk-competing antibodies compared to their uncleaved version (Figure S7d,e,g), confirming the importance of epitope cleavage. Although cleaved GPs exhibited a change in antigenicity with respect to REGN3470, all groups showed similar levels of competition with REGN3470 (Figure S7e). This discrepancy between immunogenicity and antigenicity at this site may be due to the relatively low immunogenicity of this epitope, which is reflected in the minimal competition seen in the GP-wt group. These competition experiments suggest that cleaved GPs shift antibody responses away from the glycan cap toward more conserved base and stalk epitopes, demonstrating the efficacy of our immunofocusing approach. In all, proteolytic cleavage enabled the design of EBOV-GP-based immunogens with enhanced immunogenicity to all six Ebolavirus species by targeting conserved regions on GP.

## Discussion

Designing an epitope-focused vaccine that provides broad-spectrum immunity against pathogens with high genetic variability remains a challenge. Since bnAbs may compete with strain-specific Abs and overlap in binding epitope,^5–9^ there is a need for site-specific immunofocusing techniques that can precisely modulate antibody responses. However, existing immunofocusing techniques like epitope masking techniques can dampen the immunogenicity of engineered immunogens—reducing their effectiveness.

Here, we present epitope cleavage as strategy for designing epitope-focused vaccines. Although a common approach to augment antibody responses against a given epitope is through stabilization,^23–25^ we employ the opposite strategy to both break and destabilize an undesirable epitope to shift antibody responses away from it. We first harnessed an existing method—circular permutation—by engineering new termini into the middle of the hypervariable receptor binding motif of the RBD. We found that in mice, RBDcp elicited significantly higher titers of cross-reactive antibodies than RBDwt against sarbecovirus RBDs from clades 2 and 3, and data from competition ELISA showed that RBDcp focused the antibody response away from variable epitopes targeted by class 1 and 2 antibodies onto conserved epitopes targeted by class 3 and 4 antibodies, validating the use of epitope cleavage for immunofocusing. While RBDcp did not exhibit an enhancement in cross-neutralization to related sarbecoviruses compared to RBDwt, circular permutation could be combined with mosaic display of different RBDs, which has been shown to induce broad-spectrum immunity to sarbecoviruses of different clades,^42–44^ to further enhance cross-reactive immune responses.

We also present a second protein engineering technique for epitope cleavage on a different antigen through proteolytic cleavage. Using EBOV GP and TEV protease as a model system, we inserted the TEV_CS_ into two hypervariable regions on the glycan cap of GP. We immunized mice with two cleaved immunogens (cleaved_R266_-GP and cleaved_F290_-GP) and found that both immunogens elicited higher cross-reactive titers against GPs from all six Ebolavirus GPs when compared to GPwt. We directly demonstrate that epitope cleavage—and not insertion of the TEV_CS_—alters immunogenicity, as mice immunized with cleaved GPs exhibited higher levels of cross-reactivity compared to the uncleaved GP groups. Furthermore, we found that cleaved-GPs exhibit slightly enhanced cross-neutralization to SUDV and BDBV, although future work should explore why each site only seemed to enhance neutralization against either SUDV or BDBV. While we could not construct full immunodominance hierarchies for each mouse immunized with GP variants as done previously^35^ due to serum limitations, we performed competition ELISA with an antibody panel targeting four epitopes on GP. We found that cleaved GPs steer the antibody response away from the glycan cap onto more conserved base and stalk epitopes, demonstrating the utility of proteolytic cleavage for immunofocusing.

This work provides proof-of-concept data presenting epitope cleavage as a new approach to epitope-focused vaccine design. However, the two protein-engineering approaches presented here have their own benefits and challenges. Circular permutation may be most amenable to proteins that have native N- and C-termini near each other, while proteolytic cleavage may be most suitable for larger antigens with epitopes comprised of flexible loops and could destabilize immunogens depending on the cleavage site. Furthermore, it remains to be tested if epitope cleavage can be performed on multiple sites of a protein since many antigens contain multiple immunodominant and variable sites. While we introduce epitope cleavage as a conceptual addition to the suite of existing immunofocusing approaches, other protein-engineering methods could be developed for epitope cleavage to widen the applicability of this strategy.

The serum blocking experiments performed here do not provide a full picture of the epitope landscape of the antisera elicited by our immunogens; single B-cell sorting experiments or electron microscopy polyclonal epitope mapping^45^ experiments could further elucidate the mechanistic and structural basis for how epitope cleavage enhances the cross-reactivity of antisera to diverse sarbecoviruses and Ebolavirus species. Furthermore, it will be important to investigate whether the enhancement in cross-reactivity seen in this work would affect protection in heterologous lethal challenge studies.

These data contribute to understanding the determinants of an epitope’s immunogenicity; we show that modulating an epitope’s flexibility and/or stability can decrease its immunogenicity. However, drastic changes to an epitope’s flexibility/stability seem to be required to decrease antibody responses to a given site, as simple insertion of a proteolytic site did not significantly affect the immunogenicity of our Ebola GP-based immunogens. Our work demonstrates that epitope cleavage is achievable through multiple protein engineering methods to design vaccines with enhanced cross-reactivity in two unrelated viral systems. This approach has the potential to be applied to other antigens to design effective, next-generation epitope-focused vaccines.

## Materials and Methods

### Cell Lines

HeLa-ACE2/TMPRSS2 and HEK293T-ACE2/TMPRSS2 cells were provided by Jesse Bloom at the Fred Hutchinson Cancer Research Center as a generous gift and were maintained in D10 medium—Dulbecco’s Modified Eagle Medium (DMEM, Cytiva) supplemented with 10% fetal bovine serum (GeminiBio) and 1% l-glutamine/penicillin/streptomycin (GeminiBio). HEK-293T were purchased from American type culture collection (ATCC) and maintained in D10 medium. Expi-293F cells were purchased from Thermo Fisher Scientific and maintained in Freestyle293/Expi-293 media (2:1, v/v, Thermo Fisher Scientific) in polycarbonate shaking flasks (TriForest Labware). Stellar and BL21­(DE3) competent cells were purchased from Takara Bio and Thermo Fisher Scientific, respectively.

### Antibodies

Monoclonal antibodies against SARS-CoV-2 (CB6, C002, SA58, S2X259, and SA55) and against EBOV GP (REGN3470, c13C6, mAb114, ADI-15946, c2g4, and ADI-15974) were expressed in Expi-293F cells via transient transfection. Goat antimouse IgG, HRP conjugated (BioLegend, 405306), or goat antihuman IgG, HRP conjugated (Abcam, ab98535) was used as secondary antibodies for Western blots or enzyme-linked immunosorbent assays (ELISAs).

### Antigen and Antibody Cloning

DNA encoding the EBOV GP (GenBank AAB81004.1) ectodomain with the mucin-like domain deleted (residues 1 to 308, 490 to 656) and the transmembrane domain replaced with a GCN4 ^46^ or foldon^47^ trimerization domain followed by an Avi-Tag,^48^ and a hexahistidine tag was cloned into a mammalian protein expression vector (pADD2) by In-Fusion (Takara Bio).

Similarly, DNA encoding SUDV GP (Genbank AAP88031.1) ectodomain with the mucin-like domain deleted (residues 1 to 345, 506 to 656), BDBV GP (Genbank AGL73460.1) ectodomain with the mucin-like domain deleted (residues 1 to 312, 471 to 640), RESTV GP (GenBank AAC54885.1) ectodomain with the mucin-like domain deleted (residues 1 to 308, 490 to 657), BOMV GP (GenBank MK340750.1) ectodomain with the mucin-like domain deleted (residues 1 to 304, 486 to 637), or TAFV GP (GenBank AAB37093.1) ectodomain with the mucin-like domain deleted (residues 1 to 304, 486 to 641) was cloned into pADD2 vector with a foldon trimerization domain followed by an Avi-Tag and a hexahistidine tag on the C-terminus. Uncleaved GPs were constructed by insertional mutagenesis using GPΔMLDΔfurin as the backbone.

DNA encoding SARS-CoV-2 spike (residues 1–1,143 from the Wuhan-Hu-1 genome sequence, GenBank MN908947.3) was cloned into the pADD2 vector with a GCN4 or foldon trimerization domain followed by an Avi-Tag and a hexahistidine tag on the C-terminus. DNA encoding wild-type SARS-CoV-2 Wuhan Hu-1 receptor-binding domain (residues 319–533, GenBank MN908947.3) was cloned into the pADD2 vector with a hexahistidine tag and an Avi-Tag on the C-terminus. Circularly permuted variants of RBD were cloned as previously described^49^ to generate variants with termini at each position from residues 474–503 and a (GS)_4_ linker fusing the native termini (residues 319 and 533). These constructs were cloned into the pADD2 vector with a hexahistidine tag and an Avi-Tag on the C-terminus.

Plasmids for the production of the biotinylated RBDs from WIV-1, Rf1, Yun11, BtKY72, and BM48-31 were kindly provided by the Bjorkman laboratory.

DNA fragments encoding the variable heavy chain (HC) and light chain (LC) were codon-optimized and synthesized by Integrated DNA Technologies. Fragments were inserted into an expression plasmid containing VRC01 HC and LC constant domains by In-Fusion.

All plasmids were sequence-confirmed by Sanger sequencing (MCLAB) or whole plasmid sequencing (Plasmidsaurus). For transfection purposes, plasmids were transformed into Stellar cells (Takara Bio), isolated by Maxiprep kits (Macherey Nagel or Zymo Research), filtered through a sterile 0.45-μm membrane in a biosafety cabinet, and stored at – 20 °C.

### Protein Expression and Purification

All proteins were expressed in Expi-293F cells maintained at 37 °C with constant shaking (120 rpm) in a humidified CO_2_ (8%) incubator. Expi-293F cells at a density of 3–4 × 10^6^ cells/mL were transfected using FectoPro transfection reagent (Polyplos) according to the manufacturer’s specifications. Briefly, for a 200 mL transfection, 120 μg plasmid DNA was added to 20 mL media (2:1 v/v mixture of Freestyle293 media and Expi-293 media) and vortexed after the addition of 260 μL FectoPro. The transfection mixture was allowed to incubate at room temperature for 10 min before addition to the Expi-293F cells. Immediately following transfection, cells were boosted with d-glucose (4 g/L, Sigma-Aldrich) and valproic acid (3 mM, Acros Organics). The DNA/FectoPro amount was scaled proportionally depending on the size of the transfection. For antibodies, the total DNA amount was the same but was compromised of a 1:1 mixture of heavy-chain plasmid and light-chain plasmid. Biotinylated proteins were produced by transfecting Expi-293F cells with the addition of the BirA enzyme. The transfections were harvested on day 4–5 by centrifuging the cells at 7000 g for 5 min. The resulting supernatant was filtered through a 0.22 μm membrane before further purification.

All antibodies (and ACE2-Fc) were purified by loading the filtered supernatant onto a 5 mL HiTrap MabSelect PrismA column (Cytiva) using an ÄKTA pure chromatography system (Cytiva). Post protein binding, the column was washed with HBS and then protein was eluted with 15 mL 100 mM glycine in PBS (pH 2.8) into 1.5 mL 1 M Tris pH 8.0. The eluent was then concentrated and buffer exchanged into HBS.

For His-tagged proteins, HisPur Ni-NTA resin (ThermoFisher Scientific, Cat#88221) was added to filtered supernatant (1 mL/100 mL transfection) and 2 M imidazole in HEPES buffer saline (HBS, 20 mM HEPES, pH 7.4, 150 mM NaCl) was added to the supernatant to a final concentration of 20 mM imidazole to minimize binding of impurities. Supernatant was then bound to resin overnight at 4 °C with gentle spinning. For RESTV GP and TAFV GP, supernatant was bound to resin overnight at room temperature with gentle spinning. Then, the mixture was passed over a gravity-flow column, and the collected resin was washed with 20 CV with 20 mM imidazole in HBS before eluting with 10 CV of 250 mM imidazole in HBS.

For RBDs, the elution was concentrated using AmiconR centrifugal filters (10 kDa MWCO for RBD, Millipore Sigma) and then purified on an ÄKTA pure chromatography system (Cytiva) with a Superdex 200 Increase size-exclusion chromatography column (Cytiva) in HBS. Peak fractions were collected based on the chromatography trace, measuring absorbance at 280 nm.

Spike and GPs were purified by concentrating the elution with AmiconR centrifugal filters (50 kDa MWCO for RBD, Millipore Sigma) and then purified on an ÄKTA pure chromatography system (Cytiva) with a Superose 6 Increase 10/300 GL column (Cytiva). Peak fractions were collected based on the chromatography trace, measuring absorbance at 280 nm.

For cleaved GPs, protein was mixed with TEV protease (New England Biolabs) in a 1 ug substrate to 8 uL TEV protease ratio and incubated overnight at 30 °C in a water bath. The sample was then purified on ÄKTA pure chromatography system (Cytiva) with a Superose 6 Increase 10/300 GL column (Cytiva) to remove TEV protease. Peak fractions were collected based on the chromatography trace, measuring absorbance at 280 nm.

The concentration of all proteins was determined by absorbance at 280 nm, and purity and size confirmed by protein gel electrophoresis. Protein samples were flash-frozen in PBS with 10% glycerol before for storage at −20 °C or −80 °C.

### Dot Blot

Three days after transient transfection, Expi-293F cells were harvested via centrifugation at 7,000 × g for 5 min. Two μL of supernatant was dotted directly onto a nitrocellulose membrane, left to dry for 10–15 min, and then blocked with 5% milk/H_2_O for at least 1 h or as much as overnight. For screening circular permutants of RBD, primary antibody—Rabbit anti-His Tag antibody (Proteintech, 10001-0-AP) was added for 1-h incubation, followed by donkey antirabbit IgG, HRP conjugated (1:4,000 dilution in PBST with milk) as the secondary antibody (45 min incubation). For screening GP variants with TEV_CS_ insertions, primary antibody—mAb114 (2 mg/mL, 1:4,000 dilution in PBST with milk) was then added for 1-h incubation, followed by goat antihuman IgG, HRP conjugated (1:4,000 dilution in PBST with milk) as the secondary antibody (45 min incubation). Blots were rinsed in PBST for 5 min between steps and finally developed using a Western blotting substrate (Pierce ECL, Thermo Scientific). Imaging was acquired on a chemiluminescence imager (GE Amersham Imager 600).

### Differential Scanning Fluorimetry (DSF)

Thermal melting profiles of proteins were measured by differential scanning fluorimetry on a Prometheus NT.48 instrument (NanoTemper). Protein samples (0.1 mg/mL) were loaded into glass capillaries (NanoTemper) and then subjected to a temperature gradient from 20 to 95 °C at a heating rate of 1 °C per min. Intrinsic fluorescence (350 and 330 nm) was recorded as a function of temperature. Thermal melting curves were plotted using the first derivative of the ratio (350/330 nm). Melting temperatures were calculated automatically by the instrument (PR.ThermControl software) and represented peaks in the thermal melting curves.

### Size-Exclusion Chromatography–Multi-Angle Light Scattering (SEC-MALS)

SEC-MALS analysis of immunogens was performed on a 1260 Infinity II high-performance liquid chromatography system (Agilent) coupled with a miniDAWN and Optilab detectors (Wyatt Technologies) for light scattering and refractive index analysis. Purified GPwt, GP variants (5–10 μg of each sample) were loaded onto an Superose 6 Increase 3.2/300 column sequentially for analysis. ASTRA software (Wyatt Technologies) was used for quantitative analysis of the molar mass of proteins.

### Biolayer Interferometry (BLI)

BLI experiments were performed on an Octet RED96 system (FortéBio). All samples were diluted with the Octet buffer (DPBS with 0.05% Tween 20 and 0.1% BSA), and assays were performed under agitation (1,000 r.p.m.). mAbs (CB6, C002, SA58, SA55, and S2X259, all at 200 nM) or ACE2–Fc (200 nM) were loaded onto antihuman IgG Fc capture (AHC) biosensors (FortéBio) and then dipped into antigen solutions (either RBDwt or RBDcp at 200 nM) for binding analysis, followed by dissociation into the Octet buffer. Data were processed by Data Analysis software (version 9.0.0.15, FortéBio) and then plotted.

### Antibody ELISAs

Nunc 96-well MaxiSorp plates (Thermo Fisher) were coated with streptavidin (4 μg/mL in DPBS, 60 μL per well, Thermo Fisher) for 1 h at room temperature. Plates were washed three times with Milli-Q H2O (300 μL) using a plate washer (ELx405 BioTek) and then blocked with ChonBlock (120 μL per well, Chondrex) overnight at 4 °C. For subsequent steps, all dilutions were made in DPBS with 0.05% Tween-20 and 0.1% BSA, and ELISA plates were rinsed with PBST (300 μL, three times) in between steps. Biotinylated antigen (RBDwt, RBDcp, GPwt, and GP variants at 2 μg/mL) were added to the plates and incubated for 1 h at room temperature. Then, serially diluted monoclonal antibodies (mAbs, starting from 200 nM, followed by 10-fold dilution) were added to the plates and incubated for another hour. Goat antihuman IgG, HRP-conjugated (1:4,000) was added for 1-h incubation before rinsing with PBST six times. ELISA plates were developed with the TMB substrate (1-Step Turbo-TMB, Thermo Fisher) for 6 min and terminated with sulfuric acid (2 M). Absorbance at 450 nm (A450) was recorded on a microplate reader (SynergyTM HT, BioTek).

### Mouse Immunization Studies

All animals were maintained in accordance with the Public Health Service Policy for “Human Care and Use of Laboratory Animals” under a protocol approved by the Stanford University Administrative Panel on Laboratory Animal Care (APLAC-33709). Female BALB/c mice (6 to 8 wk) were purchased from Jackson Laboratories, and female C57BL/6 mice (6 to 8 wk) were purchased from Charles River.

To compare RBDwt and RBDcp, we immunized two groups of BALB/c mice (n = 10) with 5 μg protein antigens adjuvanted with 10 μg Monophosphoryl lipid A (MPLA, Invivogen) and 10 μg Quil-A (Invivogen) via intramuscular injection on days 0, 21, 42, and 63.

To compare GPwt and cleaved GP variants, we immunized five groups of C57BL/6 mice (n = 10) with 5 μg protein antigens adjuvanted with AddaS03 (Invivogen) in a 1:1 mixture via intramuscular injection on days 0 and 21. Preimmune, interim, and final blood samples were collected by retro-orbital bleeding into serum gel tubes (Sarstedt). Serum gel tubes were centrifuged at 10,000 × g for 6 min, and sera were collected and stored at – 80 °C.

### Serum ELISAs

Nunc 96-well MaxiSorp plates were coated with streptavidin (4 μg/mL in DPBS, 60 μL per well) for 1 h at room temperature. These plates were washed three times with Milli-Q-H2O (300 μL) using a plate washer and then blocked with ChonBlock (120 μL per well) overnight at 4 °C. For subsequent steps, all dilutions were made in DPBS with 0.05% Tween-20 and 0.1% BSA, and ELISA plates were rinsed with PBST (300 μL, three times) in between. Biotinylated antigen (RBDs or GPs) (2 μg/mL) was added to the plates and incubated for 1 h at room temperature. Mouse antisera were serially diluted (5-fold dilution) and then added to the ELISA plates for 1-h incubation at room temperature. Goat antimouse IgG, HRP-conjugated (1:4,000) was added for 1-h incubation before rinsing with PBST six times. ELISA plates were developed with the TMB substrate for 6 min and terminated with sulfuric acid (2 M). Absorbance at 450 nm (A450) was recorded on a microplate reader (Synergy HT, BioTek).

For competition ELISA, competing antibodies (for RBD, CB6, C002, SA58, SA55, and S2X259 and for GP, REGN3470 c13C6, c2G4, or ADI-15974; each at 100 nM, except for REGN3470, where 800 nM was used) were preincubated with the immobilized antigen for 1 h prior to the addition of serially diluted antisera. To assess the epitope hierarchy in experiments with RBDwt and RBDcp, we followed a method to analyze competition ELISA data as previously described.^35^ In brief, titer fold-change values from duplicate experiments (performed on different days) were averaged. For competition ELISA with RBD antibodies, the titer fold changes were then normalized from 0 to 1.

### Production of Pseudotyped Lentiviruses

Sarbecovirus-spike-pseudotyped lentiviruses encoding a luciferase-ZsGreen reporter were produced in Expi293F cells by cotransfection of five plasmids. This five-plasmid system includes a packaging vector (pHAGE-Luc2-IRES-ZsGreen), a plasmid encoding full-length SARS-CoV-2 (HDM-SARS2-spike-delta21, Addgene, 155130) and three helper plasmids (pHDM-Hgpm2, pHDM-Tat1b and pRC-CMV_Rev1b). Expi-293F cells were adjusted to 3 × 106 cells per mL 1 d before transfection. For 200 mL Expi-293F cells, transfection mixture was prepared by adding five plasmids (200 μg packaging vector, 68 μg plasmid encoding the spike plasmid, and 44 μg of each helper plasmid) to 20 mL FreeStyle293/Expi-293 media, followed by the addition of 600 μL BioT transfection reagent in a dropwise manner with vigorous mixing. After 10 min incubation at room temperature, the transfection mixture was transferred to Expi-293F cells. Expi-293F cells were immediately boosted with d-glucose (4 g/L) and valproic acid (3 mM). After 72 h, viruses were harvested and filtered through a 0.45-μm membrane.

BtKY72 (K493Y/T498W) lentivirus was concentrated using Lenti-X Concentrator (Takara) using the manufacturer’s protocol to a 20x volume. The virus was then aliquoted, flash-frozen in liquid nitrogen, stored at – 80 °C, and titrated in HEK293T-ACE2/TMPRSS2 cells before further use.

All SARS-CoV-2, SARS-CoV-1, and WIV1 lentiviruses were not concentrated but were stored with the same method as described above and titrated in HeLa-ACE2/TMPRSS2 cells before further use.

EBOV, SUDV, and BDBV GP-pseudotyped lentiviruses encoding a luciferase-ZsGreen reporter were produced using the same method but with plasmids encoding GPs (pcDNA3.1 FL-GP) swapped for spike-encoding plasmids.

### Serum Neuralization Assays against Pseudoviruses

Antisera were heat inactivated (56 °C, 30–60 min) before neutralization assays. Neutralization against Ebolavirus GPs was analyzed in HEK293T cells with mouse antisera against Ebola GP. Neutralization against sarbecovirus pseudoviruses was analyzed in HeLa-ACE2/TMPRSS2 cells or HEK293T-ACE2/TMPRSS2 with mouse antisera against RBD. In brief, cells were seeded in white-walled, clear-bottom, 96-well plates 1 d before the assay (day 0) at a density of 8,000 cells/well for HeLa-ACE2/TMPRSS2, 40,000 cells/well for HEK293T-ACE2/TMPRSS2, and 20,000 cells/well for HEK293T. On day 1, antisera were serially diluted in D10 media and mixed with pseudoviruses for 1 h before being transferred to cells. Assays against Ebolavirus or sarbecovirus pseudoviruses were read out with luciferase substrates 3 days or 2 days after infection, respectively. Percent infection was normalized on each plate. NT50 were calculated as the serum dilution where a 50% inhibition of infection was observed. Neutralization assays were performed in technical duplicates.

### Statistics and Reproducibility

Dot blot analysis and serological analyses were repeated at least twice with similar results, and one set of representative data are presented. Statistics were analyzed using GraphPad Prism software (version 10.6.1). Nontransformed data are presented as mean ± s.d. ELISA titers and NT_50_ were log10-transformed and presented as geometric mean ± s.d. Comparisons of two groups were performed using the two-tailed Mann–Whitney U-test. P values of 0.05 or less are considered significant.

## Supplementary Material



## Data Availability

Further information and requests for resources and reagents should be directed to and will be fulfilled by the lead contact, Peter S. Kim (kimpeter@stanford.edu).

## Notes

BLI binding curves for RBDcp and RBDwt, immunogenicity and competition ELISA data for mouse immunizations, neutralization curves, biochemical characterization of GP ΔMLDΔfurin, dot blot of GPs with TEV_CS_ insertion, melting temperatures and SEC-MALS of cleaved GP variants, and antigen sequences (PDF)
