# Supplementary material for: Design of Epitope-Focused Vaccines via Epitope Cleavage
Source: ACS Cent Sci. 2026 Jul 17;12(9):1348–59. doi: 10.1021/acscentsci.6c00476 (PMC13614085; doi:10.1021/acscentsci.6c00476)
Supplement: Supplementary file 1 [file oc6c00476_si_001.pdf]

Supporting Information

Design of epitope-focused vaccines via epitope cleavage

Dominic M. Pham,[1,2] Rebekah M. Costello,[2,3] Theodora U. J. Bruun, [2,4] Peter S. Kim*[2,4]
[1]Stanford Biophysics Program, Stanford University School of Medicine; Stanford, CA 94305, USA
[2]Sarafan ChEM-H, Stanford University; Stanford, CA 94305, USA
[3]Program in Immunology, Stanford University School of Medicine; Stanford, CA 94305, USA
[4]Department of Biochemistry, Stanford University School of Medicine; Stanford, CA 94305, USA
*Corresponding author. Email: kimpeter@stanford.edu

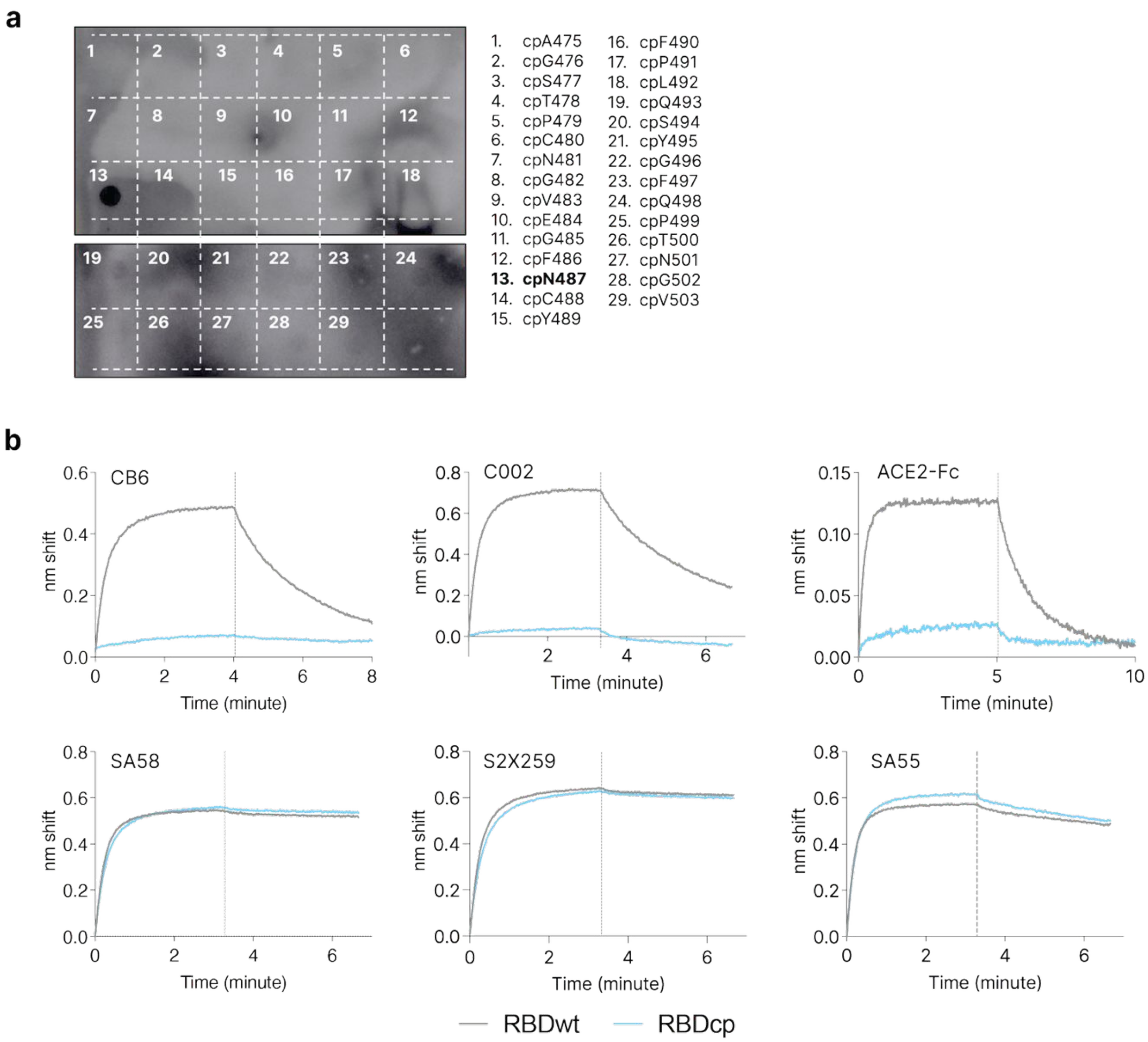

Figure S1. Screening circular permutants of RBD and antigenicity of RBDcp. a, Dot-blot to determine the expression levels of circular permutants of RBD. Blot was blocked and incubated with rabbit anti-His Tag antibody and detected with HRP-conjugated donkey anti-rabbit IgG. **b,** BLI binding profiles of wild-type or circularly permuted RBD with five RBD-specific mAbs (CB6, C002, SA58, S2X259, and SA55) and ACE2-Fc. Vertical dashed lines indicate the beginning of dissociation.

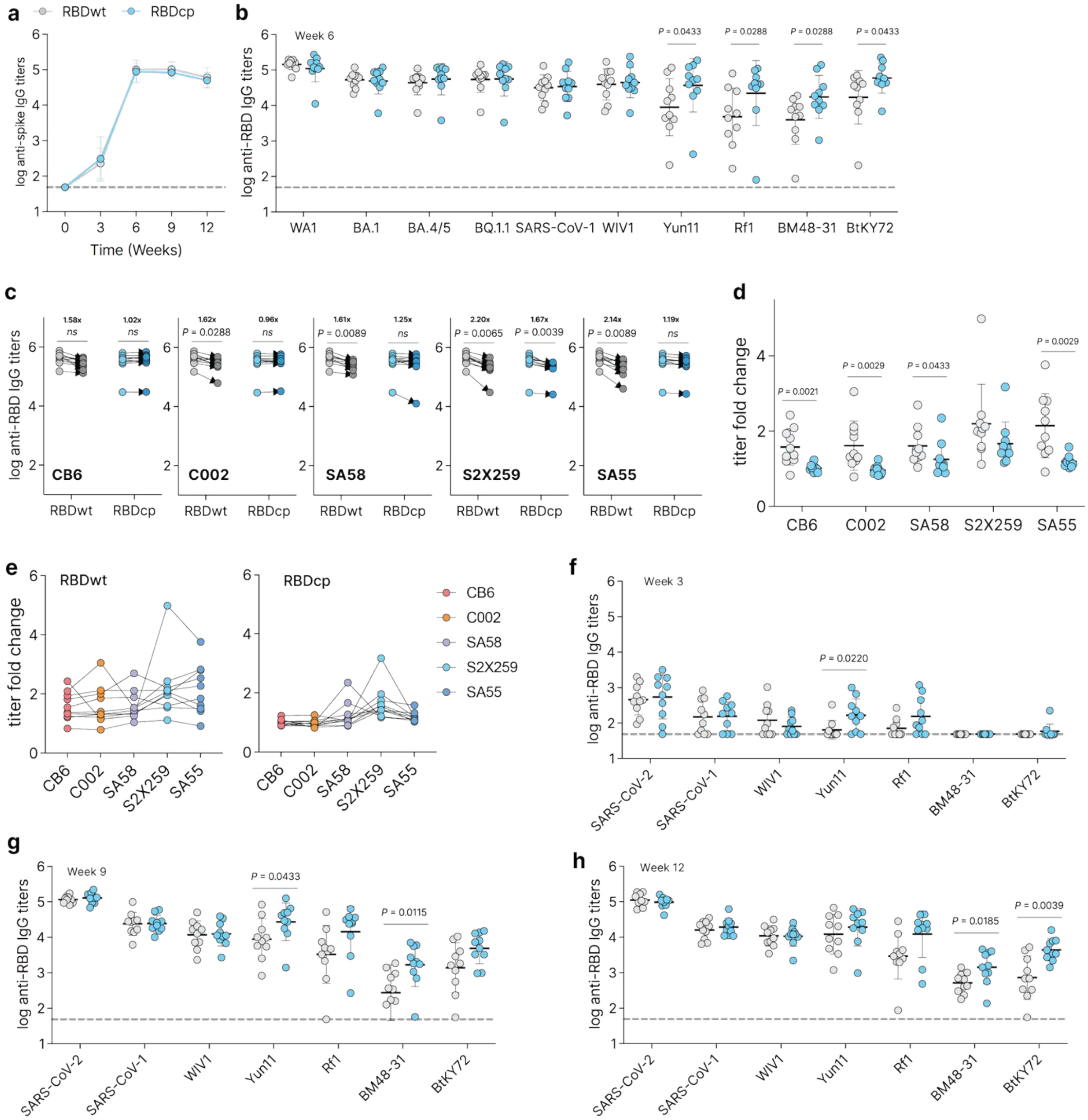

Figure S2. Immunogenicity of RBDwt and RBDcp. a, Serum IgG titers against SARS-CoV-2 6P Spike over time. Each circle represents the geometric mean IgG titer per group. **b,** IgG titers against SARS-CoV-2 variants (WA1, BA.1, BA.4/5, and BQ.1.1), clade 1a (SARS-CoV-1 and WIV1), clade 2 (Yun11 and Rf1), and clade 3 (BM48-31 and BtKY72) sarbecovirus RBDs by week 6 antisera. Each circle represents a single mouse. **c,** IgG titers before and after epitope blocking with RBD-targeted antibodies. Geometric mean titer fold-changes are shown above each column. **d,** Titer fold-changes from competition ELISA with week 6 serum in the presence of competing mAbs (CB6, C002, SA58, S2X259, SA55). Each circle represents the titer fold-change from a single mouse (n=10 mice per group). **e,** Individual IgG titer fold-changes after epitope blocking with five RBD-specific mAbs for the RBDwt (left) and RBDcp (right) groups. Each circle represents the titer fold-change corresponding to a given antibody for a single mouse. Lines connect titer fold-changes for a single mouse. **f, g, h,** IgG titers against clade 1a (SARS-

CoV-1 and WIV1), clade 2 (Yun11 and Rf1), and clade 3 (BM48-31 and BtKY72) sarbecovirus RBDs by week 3 **(f),** week 9 **(g),** and week 12 **(h)** antisera. Each circle represents a single mouse.

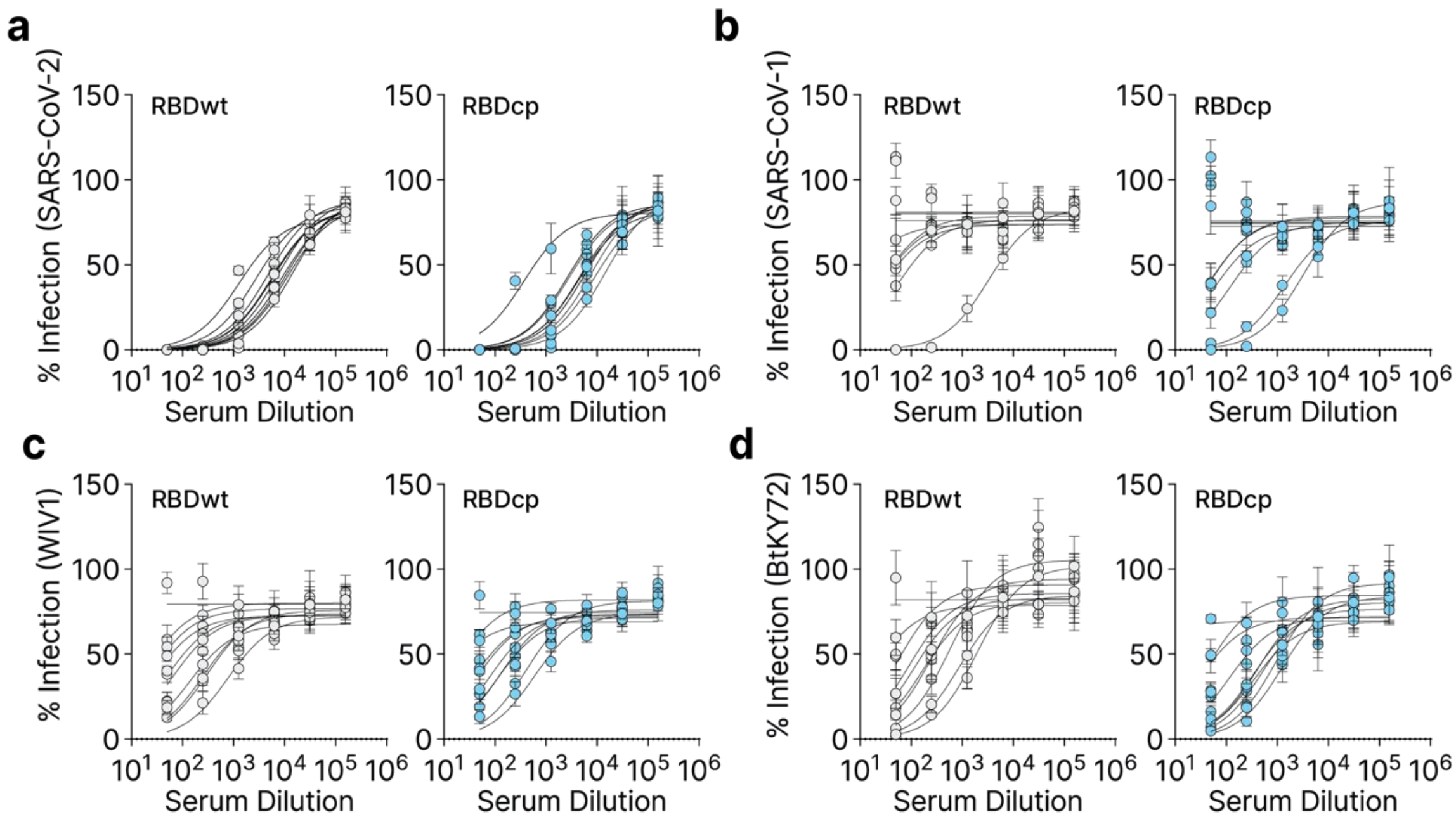

Figure S3. Neutralizing antibody responses to RBDwt and RBDcp. Serum neutralization profiles of (**a**) SARS-CoV-2 WA.1, (**b**) SARS-CoV-1, and (**c**) WIV1 and (**d**) BtKY72 pseudoviruses at week 12. Each curve is derived from a single mouse (n = 10). Data are presented as mean ± s.d. of technical duplicates in neutralization curves.

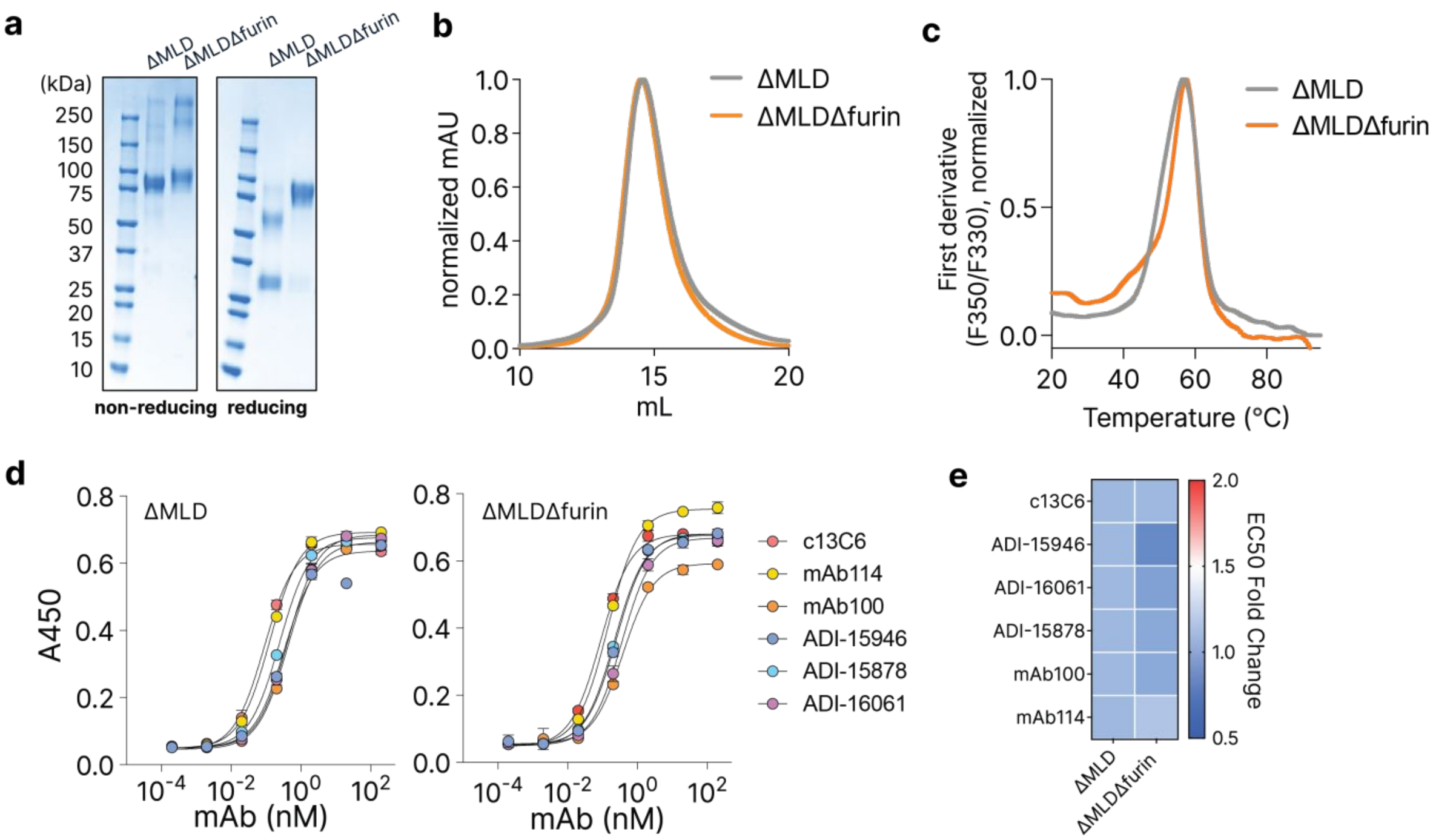

Figure S4. Mutating furin cleavage site does not affect stability or antigenicity of EBOV GP. a, Gel electrophoresis of wild-type and Δfurin GP. Gel was run under nonreducing (left) and reducing (right) conditions and stained with Coomassie brilliant blue. **b,** Size-exclusion purification of ΔMLD and ΔMLDΔfurin GP. **c,** Representative thermal melting profiles and melting temperatures of ΔMLD and ΔMLDΔfurin GP measured by differential scanning fluorimetry. **d,** ELISA for epitope analysis of ΔMLD and ΔMLDΔfurin GP with six GP-specific mAbs. Binding data are presented as mean ± SD (n = 3 technical replicates). **e,** Fold-change in binding (EC_{50}) of GP-specific mAbs (columns) to ΔMLD and ΔMLDΔfurin GP (rows). EC_{50} (antibody concentration with half-maximal binding) was calculated from (**d**) and normalized to values obtained from the ΔMLD group.

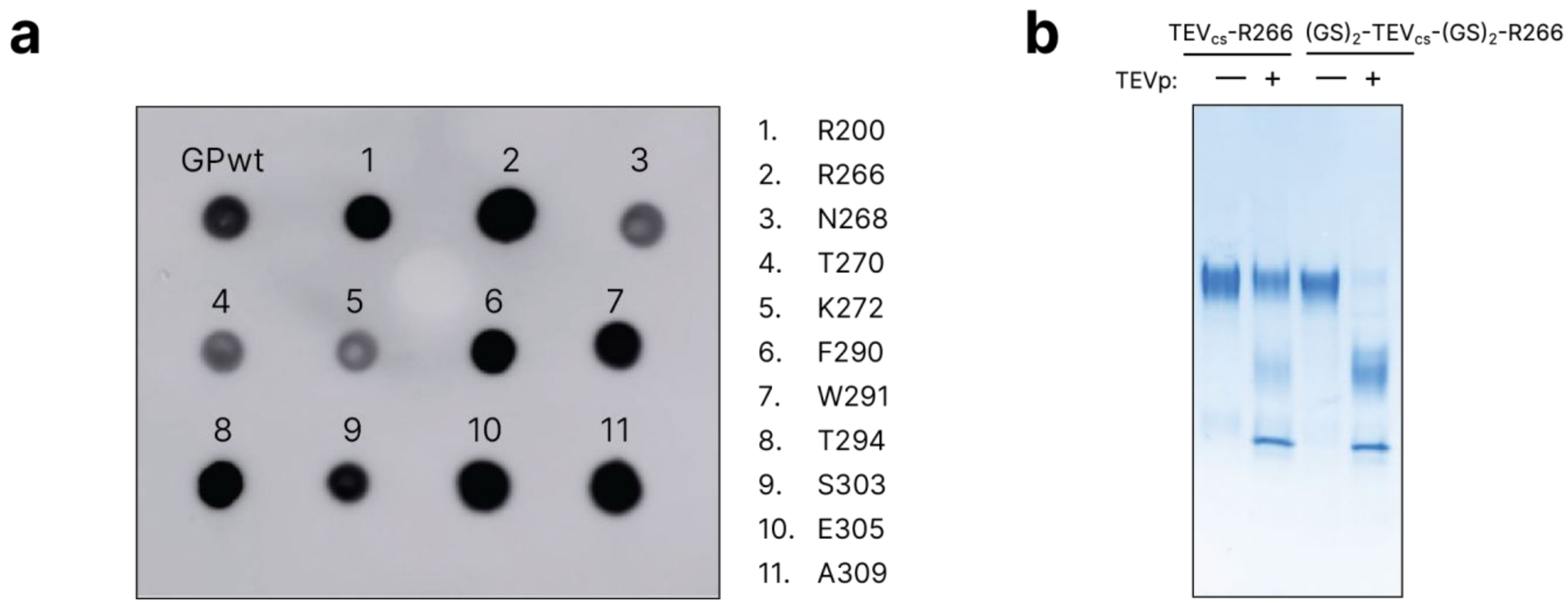

Figure S5. Screening and optimization of cleaved GP designs. a, Dot-blot to determine the expression levels of GP with TEV_{CS} insertion at 11 distinct sites on the glycan cap. Blot was blocked and incubated with mAb114 and detected with HRP-conjugated goat anti-human IgG. **b,** Gel electrophoresis of GP with the R266 TEV_{CS} insertion and the TEV_{CS} insertion flanked by $(GS)_2$ linkers. Gel was run under reducing conditions and stained with Coomassie brilliant blue.

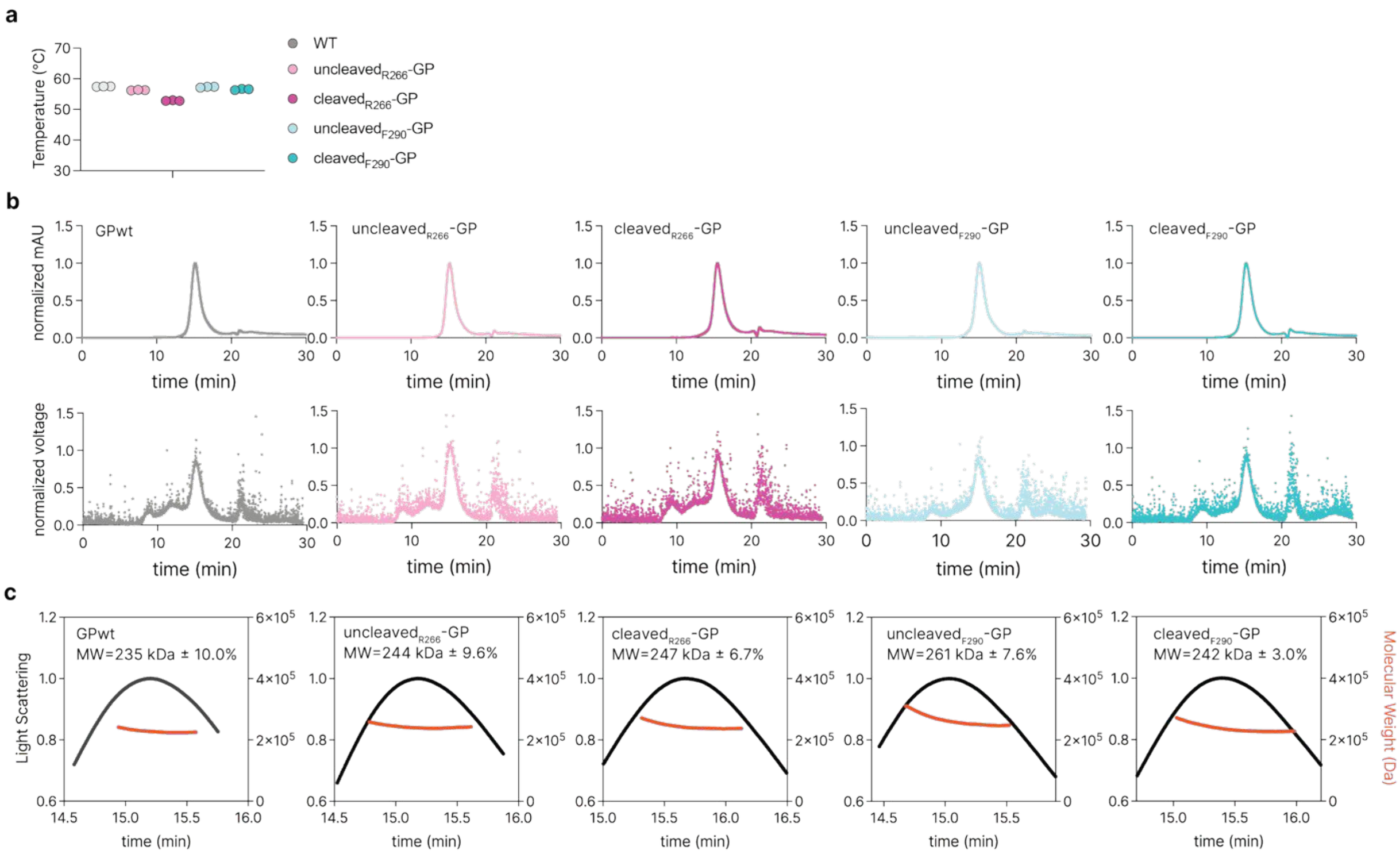

Figure S6. Biochemical characterization of cleaved GP variants. a, Melting temperatures of cleaved GP variants measured by DSF. **b,** Size-exclusion chromatography coupled with multi-angle light scattering (SEC-MALS) analysis of GP variants on a Superose® 6 Increase 10/300 GL column. mAU and voltage indicate absorbance at 280 nm and light scattering signals, respective. **c,** Light scattering profiles and molecular weight of GPwt and cleaved GP variants calculated from the refractive indices and elution profiles using ASTRA software.

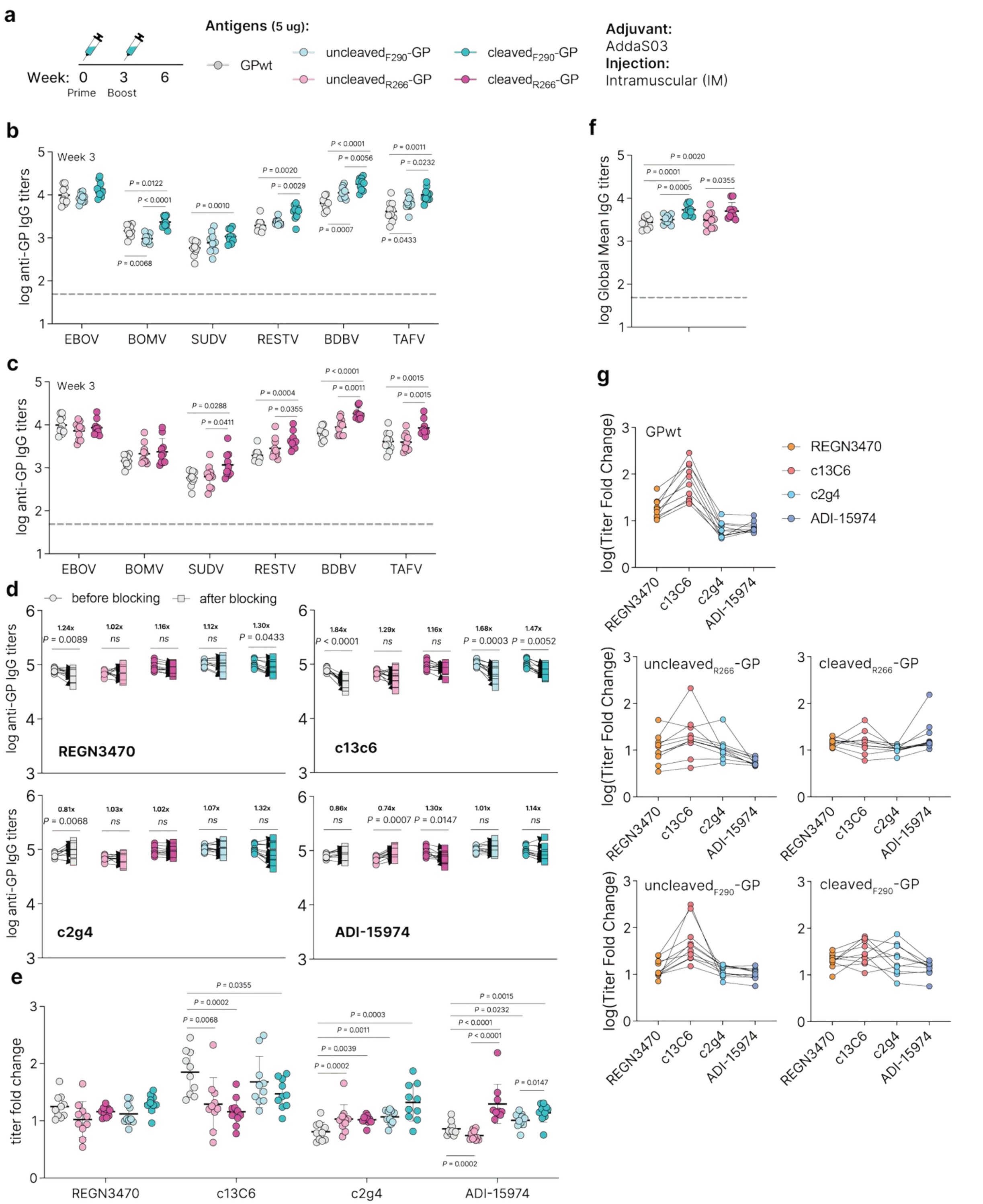

Figure S7. Immunogenicity of EBOV GP and cleaved GPs variants. a, A two-dose immunization study with GPwt, uncleaved$_{R266}$-GP, cleaved$_{R266}$-GP, uncleaved$_{F290}$-GP, and cleaved$_{F290}$-GP adjuvanted with AddaS03 via intramuscular injection in mice. **b,c,** IgG binding titers against GPs from all six ebolavirus species (EBOV, BOMV, SUDV, RESTV, BDBV and TAFV) by week 3 antisera for groups immunized with uncleaved$_{R266}$-GP and cleaved$_{R266}$-GP (**b**)

or uncleaved$_{F290}$-GP and cleaved$_{F290}$-GP (**c**). Each circle represents a single mouse (n=10 mice per group). **d,** IgG titers before and after epitope blocking with GP-targeted antibodies. Geometric mean titer fold-changes are shown above each column. **e,** Titer fold-changes from competition ELISA with week 6 serum in the presence of competing mAbs (REGN3470, c13C6, c2g4, and ADI-15974). Each circle represents the titer fold-change from a single mouse (n=10 mice per group). **f,** Global geometric mean titers against all 6 GPs tested at week 3. Each circle represents a single mouse (n=10 mice per group). **h,** Individual IgG titer fold-changes after epitope blocking with four GP-specific mAbs. Each circle represents the titer fold-change corresponding to a given antibody for a single mouse. Lines connect titer fold-changes for a single mouse. The data for GPwt, cleaved$_{R266}$-GP, and cleaved$_{F290}$-GP is the same as that presented in **Fig. 5.**

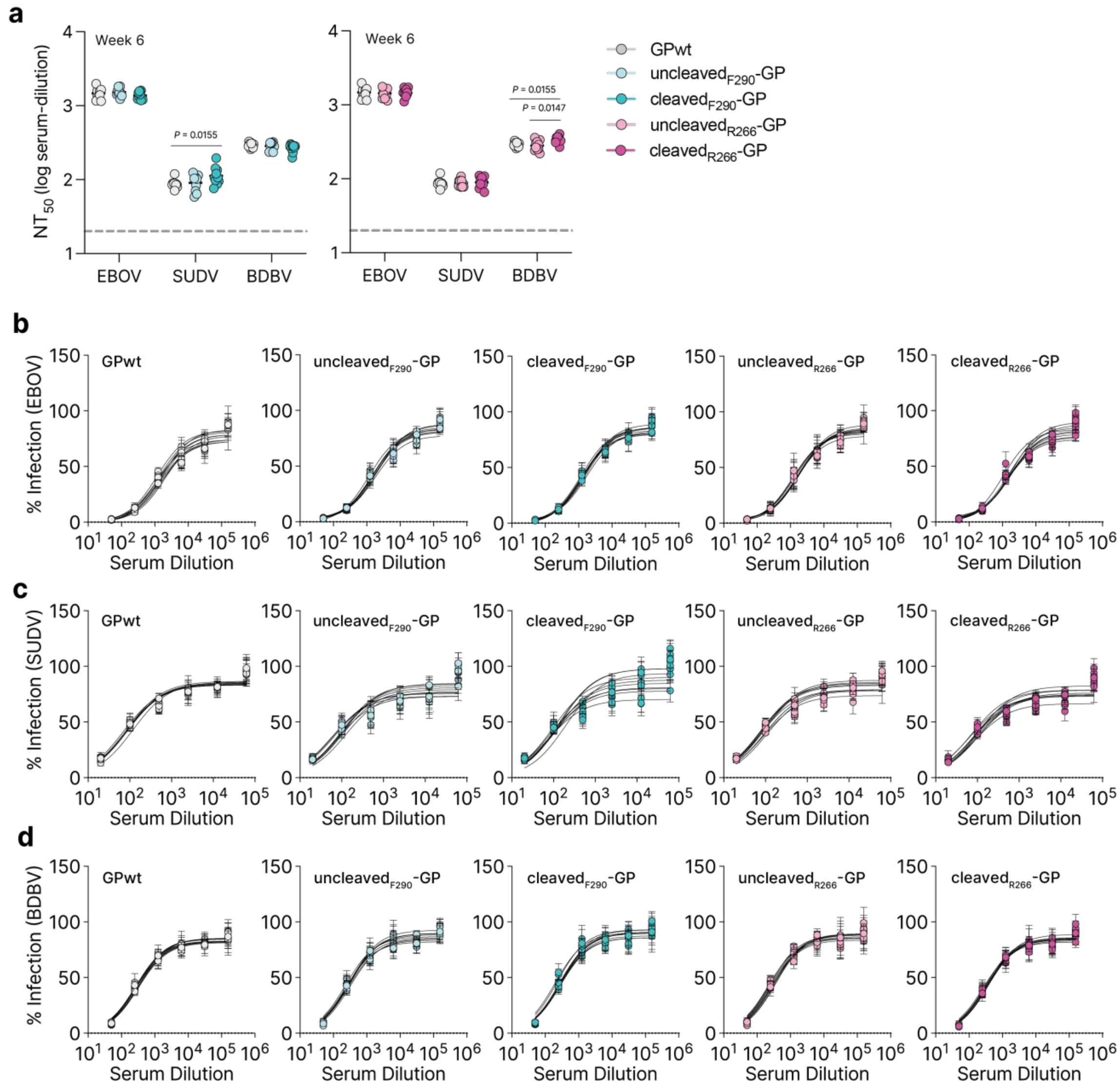

Figure S8. Neutralizing antibody responses to EBOV GP and cleaved GP variants. a, Neutralization titers against EBOV, SUDV and BDBV pseudotyped viruses by week 6 antisera for groups immunized with uncleaved$_{R266}$-GP and cleaved$_{R266}$-GP (left) or uncleaved$_{F290}$-GP and cleaved$_{F290}$-GP (right). **b,c,d,** Serum neutralization profiles of (**b**) EBOV, (**c**) BDBV and (**d**) SUDV pseudoviruses. Each curve is derived from a single mouse (n = 10). Data are presented as mean ± s.d. of technical duplicates in neutralization curves. The data for GPwt, cleaved$_{R266}$-GP, and cleaved$_{F290}$-GP is the same as that presented in **Fig. 5.**

Antigen Sequences

6x Histidine Tag (boldfaced residues)
Avi-Tag (underlined residues)
TEV site (marked in grey)

SARS-CoV-2 RBD (RBDwt)

RVQPTESIVRFPNITNLCPFGEVFNATRFASVYAWNRKRISNCVADYSVLYNSASFSTFK
CYGVSPTKLNDLCFTNVYADSFVIRGDEVRQIAPGQTGKIADYNYKLPDDFTGCVIAWN
SNNLDSKVGGNYNYLYRLFRKSNLKPFERDISTEIYQAGSTPCNGVEGFNCYFPLQSYGF
QPTNGVGYQPYRVVVLSFELLHAPATVCGPKKSTNLG**HHHHHH**GGLNDIFEAQKIEWH
E

RBDcp

CYFPLQSYGFQPTNGVGYQPYRVVVLSFELLHAPATVCGPKKSTNLGSGSGSGSRVQPT
ESIVRFPNITNLCPFGEVFNATRFASVYAWNRKRISNCVADYSVLYNSASFSTFKCYGVS
PTKLNDLCFTNVYADSFVIRGDEVRQIAPGQTGKIADYNYKLPDDFTGCVIAWNSNNLD
SKVGGNYNYLYRLFRKSNLKPFERDISTEIYQAGSTPCNGVEGFNG**HHHHHH**GGLNDIF
EAQKIEWHE

Ebola Glycoprotein (GPwt)

SIPLGVIHNSTLQVSDVDKLVCRDKLSSTNQLRSVGLNLEGNGVATDVPSATKRWGFRS
GVPPKVVNYEAGEWAENCYNLEIKKPDGSECLPAAPDGIRGFPRCRYVHKVSGTGPCA
GDFAFHKEGAFFLYDRLASTVIYRGTTFAEGVVAFLILPQAKKDFFSSHPLREPVNATED
PSSGYYSTTIRYQATGFGTNETEYLFEVDNLTYVQLESRFTPQFLLQLNETIYTSGKRSNT
TGKLIWKVNPEIDTTIGEWAFWETKKNLTRKIRSEELSFAGLITGGAGTAAEAIVNAQPK
CNPNLHYWTTQDEGAAIGLAWIPYFGPAAEGIYIEGLMHNQDGLICGLRQLANETTQAL
QLFLRATTELRTFSILNRKAIDFLLQRWGGTCHILGPDCCIEPHDWTKNITDKIDQIIHDFV
DKTLPDQGDNDNWWTGWRQWIPAGIMKQIEDKIEEILSKIYHIENEIARIKKLIGEVASSS
GLNDIFEAQKIEWHEA**HHHHHH**G

Uncleaved$_{R266}$-GP

SIPLGVIHNSTLQVSDVDKLVCRDKLSSTNQLRSVGLNLEGNGVATDVPSATKRWGFRS
GVPPKVVNYEAGEWAENCYNLEIKKPDGSECLPAAPDGIRGFPRCRYVHKVSGTGPCA
GDFAFHKEGAFFLYDRLASTVIYRGTTFAEGVVAFLILPQAKKDFFSSHPLREPVNATED
PSSGYYSTTIRYQATGFGTNETEYLFEVDNLTYVQLESRFTPQFLLQLNETIYTSGKRGSG
SENLYFQSGSGSSNTTGKLIWKVNPEIDTTIGEWAFWETKKNLTRKIRSEELSFAGLITGG
AGTAAEAIVNAQPKCNPNLHYWTTQDEGAAIGLAWIPYFGPAAEGIYIEGLMHNQDGLI
CGLRQLANETTQALQLFLRATTELRTFSILNRKAIDFLLQRWGGTCHILGPDCCIEPHDW
TKNITDKIDQIIHDFVDKTLPDQGDNDNWWTGWRQWIPAGIMKQIEDKIEEILSKIYHIEN
EIARIKKLIGEVASSSGLNDIFEAQKIEWHEA**HHHHHH**G

Uncleaved$_{F290}$-GP

SIPLGVIHNSTLQVSDVDKLVCRDKLSSTNQLRSVGLNLEGNGVATDVPSATKRWGFRS
GVPPKVVNYEAGEWAENCYNLEIKKPDGSECLPAAPDGIRGFPRCRYVHKVSGTGPCA
GDFAFHKEGAFFLYDRLASTVIYRGTTFAEGVVAFLILPQAKKDFFSSHPLREPVNATED
PSSGYYSTTIRYQATGFGTNETEYLFEVDNLTYVQLESRFTPQFLLQLNETIYTSGKRSNT
TGKLIWKVNPEIDTTIGEWAFENLYFQSWETKKNLTRKIRSEELSFAGLITGGAGTAAEAI
VNAQPKCNPNLHYWTTQDEGAAIGLAWIPYFGPAAEGIYIEGLMHNQDGLICGLRQLA
NETTQALQLFLRATTELRTFSILNRKAIDFLLQRWGGTCHILGPDCCIEPHDWTKNITDKI
DQIIHDFVDKTLPDQGDNDNWWTGWRQWIPAGIMKQIEDKIEEILSKIYHIENEIARIKKL
IGEVASSSGLNDIFEAQKIEWHEA**HHHHHH**G
